# Global and regional prevalence of *Cronobacter sakazakii* in powdered milk and flour

**DOI:** 10.1038/s41598-024-57586-x

**Published:** 2024-03-22

**Authors:** Temitope C. Ekundayo, Oluwatosin A. Ijabadeniyi

**Affiliations:** Department of Biotechnology and Food Science, Durban University of Technology, Steve Biko Campus, Steve Biko Rd, Musgrave, Berea, Durban, South Africa

**Keywords:** Sample size, Detection methods, DNA extraction, Powdered infant formula, Applied microbiology, Bacteria, Microbiology, Industrial microbiology

## Abstract

*Cronobacter sakazakii* (Cz) infections linked with powdered milk/flour (PMF) are on the increase in recent times. The current study aimed at assessing worldwide and regional prevalence of Cz in PMF. Cz-PMF-directed data were conscientiously mined in four mega-databases via topic-field driven PRISMA protocol without any restriction. Bivariate analysis of datasets was conducted and then fitted to random-intercept logistic mixed-effects regressions with leave-one-study-out-cross-validation (LOSOCV). Small-study effects were assayed via Egger’s regression tests. Contributing factors to Cz contamination/detection in PMF were determined using 1000-permutation-bootstrapped meta-regressions. A total of 3761 records were found out of which 68 studies were included. Sample-size showed considerable correlation with Cz positivity (r = 0.75, *p* = 2.5e−17), Milkprod2020 (r = 0.33, *p* = 1.820e−03), and SuDI (r = − 0.30, *p* = 4.11e−03). The global prevalence of Cz in PMF was 8.39% (95%CI 6.06–11.51, PI: 0.46–64.35) with LOSOCV value of 7.66% (6.39–9.15; PI: 3.10–17.70). Cz prevalence in PMF varies significantly (*p* < 0.05) with detection methods, DNA extraction method, across continents, WHO regions, and world bank regions. Nation, detection method, world bank region, WHO region, and sample size explained 53.88%, 19.62%, 19.03%, 15.63%, and 9.22% of the true differences in the Cz prevalence in PMF, respectively. In conclusion, the results indicated that national will power in the monitoring and surveillance of Cz in PMF matched with adequate sample size and appropriate detection methods will go a long way in preventing Cz contamination and infections.

## Introduction

*Cronobacter* is currently a seven-species member Gram-negative bacterial genus. They are peritrichously flagellated, motile, bacillary shaped, nonspore-forming facultative anaerobes belonging to the family *Enterobacteriaceae*^1,2^. The seven species that comprised the genus include *C. condiment, C. dublinensis*, *C. malonaticus*, *C. muytjensii*, *C. sakazakii*, *C. turicensis*, and *C. universalis*^3^. *Cronobacter* species is an emerging food- and environmental borne pathogen^4^. It is notably linked with powdered infant formula (PIF), milk and dairy products, drinking water, vegetables, meat, cereals, herbs, and spices^5–10^. The pathogen has been isolated from a variety of food and environmental matrices including tap water, underground water, river water^6^, aquatic products^11^, flours^12^, meat products^13^, cereal products^14,15^, read-to-eat foods^14,15^, food animals’ offal^16^, and other matrices. The desiccation-tolerance/resistance and biofilm production capability of *Cronobacter* offered it exceptional ability to attached to and survive surfaces of packaging materials, equipment, and utensils in food production facilities and farm-environments^16–18^. Thus, *Cronobacter*’s contamination constitutes a menace in food processing especially dairy and meat industries.

*Cronobacter* spp. are generally regarded as significant health threats in children, the immunosuppressed and immunocompromised individuals. *Cronobacter* infections often lead to life-threatening disease conditions in the paediatrics, elderly and immunocompromised patients including meningitis, sepsis, bacteraemia, and necrotizing enterocolitis with previous reported death rate exceeding 40–80%^19,20^. Additionally, there were reports of acute, long-term, and chronic sequelae in *Cronobacter* infection survivals such as brain abscesses, quadriplegia, hydrocephalus, neural-development delay, and other neurological complications^21,22^. Hence, there is a need for more awareness campaign to spur significant research efforts on the control and prevention of *Cronobacter* for sustainable paediatric food safety, neonatal and public health.

Further, *Cronobacter* can survive and escape the available decontamination process conditions including the use of exogenic detergents, chemical disinfectants such as hydrogen peroxide and sodium hypochlorite^23^, ultraviolet irradiation and near-infrared irradiation^24^ in food and dairy industries, making them among neglected emerging biohazards in widely distributed finished products. This is partly due to the ability of *Cronobacter* to produce biofilms and develop resistance to exogenic detergents and chemical disinfectants and jointly, due to ability of the chemical disinfectants, ultraviolet irradiation, and near-infrared irradiation at required operational doses employed to induce resistance-favouring mutations in the pathogen^22,25,26^. Further, antibiotic resistance is rapidly increasing in *Cronobacter*^27^. Thus, research into new effective, efficient, inexpensive, safe, and sustainable antibiotics and methods for decontaminating *Cronobacter* in food processing and environmental facilities and treatment of its infections are required. Therefore, comprehensive assessment of the prevalence of Cz is required to identify knowledge gaps to drive new research focuses.

Regardless of the food safety, paediatric and the immunocompromised health concerns of *Cronobacter*, the surveillance of *Cronobacter* spp. in PIF, milk, finished products and food processing facilities/environment have not received deserved attention in most countries. Therefore, considering the continuous increase in immunocompromised health conditions, increasing and rapid antibiotic resistance and high paediatric prevalence of *Cronobacter* infections, as well as associated high fatality rate and sequelae worldwide, there is a crucial need to assess the global and regional prevalence of *Cronobacter* and the associated factors governing its prevalence in powdered milk/flour (PMF). For this purpose, this study aims to assess the global prevalence of Cz and its affinity with technical procedures and regional socioeconomic statuses.

## Materials and methods

### Data source and selection

*Cronobacter sakazakii* (*Cz*) data associated with PMF contamination were conscientiously retrieved without any restrictions from mega repositories hosting quality peered reviewed studies (EBSCOhost (including CINAHL, MEDLINE, APA PsycInfo, SocINDEX, CAB Abstracts, SPORTDiscus, GreenFILE, Global Health etc.), WoS (Web of Science), PubMed, and Scopus) from inception to 2023 using “(Cronobacter* OR sakazakii) AND (flour* OR powder* OR milk* OR formula*)” and its variant specification according to different databases’ allowable algorithms for primary research articles. The first and second part of the query was executed as title-specific and topic-specific search in the combination (details in appendix) based on PRISMA version 2020 (“Preferred Reporting Items for Systematic Reviews and Meta-analyses)”^28^ respectively. Data acquisition was first attempted on 07 March 2023 11:40:09 A.M. and followed with a final update to include update from database inception till 31 December 2023.

### Data inclusion and exclusion criteria

*Cz*-PMF specific studies were adjudged eligible if *Cz* was the targeted outcome irrespective of the detection means. The following details are also essential for study’s inclusion and rating: study’s descriptors (authors, sampling plan/sample size), methodologic elements (sample preparation and detection techniques), and outcome (*Cz* positivity/negativity records). PMF in this current study referred to pulverised grain/milk (usually characterised with low water activities). For a study to be included, it must report number of *Cz-*specific positive samples, PMF-specific (or its subdivision as described in section “Data treatments") sample size collected, *Cz* isolation method, Cz confirmation strategy (cultural, serological, PCR, and DNA extraction technique). Studies or sub-sample categories that reported sample size < 10 were excluded. Any study lacking one or more of the study descriptors, methodologic elements, and Cz-specific outcome were excluded. Also, laboratory stimulated/studies with spike samples, editorials, opinions, and reviews articles were excluded.

### Data treatments

An aggregate of 26,142 *Cz*-PMF studies identified from the databases was de-duplicated in Endnote version 20 and Excel version 2016. TE screened a total of 80 unique articles following the de-duplication by titles/abstracts. Of these, only 68 studies fulfilled the eligibility criteria and with potential desirable data indications were downloaded for data extraction (supplementary materials). TE and an outsourced consultant reviewer extracted the data and populated pre-designed table forms with the extracted data in 3 separate efforts designated as sets (Cza, Czb, and Czc) and validated (Eq. 1) for further analysis. The data included study’s identity (first author’ name and year (PY)), sample size (K), PMF type, *Cz-*positivity record (P), detection method, and national affiliation.

1 $$  \left( {\left| {{\text{Cza}} \cup {\text{Czb}} \cup {\text{Czc}}} \right|} \right)/\left( {\left| {{\text{Cza}} \cap {\text{Czb}} \cap {\text{Czc}}} \right|} \right) \equiv 1  $$

Further data validation was carried out by co-author (OA) and an outsourced consultant reviewer. Disagreements were resolved by discussion. Studies’ qualities were assessed as presented in the supplementary material. Studies with > 1 category/type of samples were further disaggregated to the respective components with their prevalence estimate recorded or calculated from the corresponding data. The data was disaggregated based on sample type into IFF (infant formula flour (IFF)/Flour: flour/instant dry soup samples/wheat-based infant food, potato dumpling powder; dehydrated rice powder (DRP), infant rice powder (IRF); breast milk substitutes (corn starch, plantain starch, other starches); IMF//powdered porridges/custards; soy-based infant formulae), PIF (powdered milk-based PIF (powdered infant formula): prefinal product/prepackaged final product/final packaged product/; infant formula milk powder (IFMP), dried milk, FUF (follow up formula)/dried milk (Full-fat milk powder, skimmed milk powder, dried whey, dried ice-cream, dried artificial cream Sahlab, Infant milk formulas; milk powder; FUF, powder adult formula (PAF//CPIF), Ifoods (infant foods), CPIF (cereal-based powdered weaning food products/cereal based infant formulas and complementary foods, cereal mixes for children; corn-based farinaceous food), and EPIF (environ. Samples from PIF factories; infant formulae factories, dust; goat powder milk facilities, environmental sample of milk powder manufacturing facilities, utensils, bottles containing thickened cow’s milk, used feeding bottles, bottle brushes, dosing cups, bottle storage equipment and blenders).

Additional countries and regional data such as World Bank Country and Lending Groups (WB income)(https://datahelpdesk.worldbank.org/knowledgebase/articles/906519-world-bankcountry-and-lending-groups), Human Development Index (HDI2021) by Country 2022.

(https://worldpopulationreview.com/country-rankings/hdi-by-country), world milk production (Milkprod2020) (https://ourworldindata.org/grapher/milk-production-tonnes), WHO region (https://www.greenfacts.org/glossary/wxyz/who-regions.htm), Sustainable Development Index (SuDI) (https://www.sustainabledevelopmentindex.org/), and Socio-Demographic Index (SDI)/(SDI quintile) (GBD, 2020) were assessed and retrieved on 29 March 2023.

### Statistical analysis

A total of 23,106 *Cz*-PMF dataset were extracted and disaggregated into IFF (Infant formula flour/flour); PIF (powdered infant milk-based formula), Ifoods (infant foods), CPIF (cereal-based powdered weaning food products/cereal based infant formulas and complementary foods), and EPIF (PIF taken from infant formulae factories’ environments). First, the whole data was subjected to descriptive analysis. Bivariate analyses were also conducted to explore associations among Cz positivity records, sample size and region-specific data (Milkprod2020, WB income, HDI2021, Milkprod2020, and SuDI). Then the Cz proportion (p/n) was logit normalized^29^ and fitted to a random intercept logistic regression (RILR) coupled with continuity correction by a 0.5 to account for individual study with zero frequencies. The 95% confidence interval (95%CI) of the random effects in the RILR was estimated based on t-distribution. The between-study heterogeneity (I^2^ & H^2^) was derived via maximum-likelihood estimator and an I^2^ test ≥ 75% was signified as considerable heterogeneity^30^. Small-study effects/bias were tested via Egger’s regression tests^31^ and model’s stability established by using leave-one-study-out-cross-validation (LOSOCV)^32^. LOSOCV involved recalculation of pooled prevalence effect estimate with one study omitted each time in order to identify and remove outlying case(s) or influential case(s). A significant intercept (*p* ≤ 0.05) Egger’s regression indicates presence of bias and vice versa.

Detection method, PMF type, nation, and regional data/designations were utilized in a mixed-effects RILR sub-group analyses where within-group prevalence and subgroup differences was assayed via a random-effects-model and a common-effects-model respectively^30^. Furthermore, the RILR estimates was subjected to either univariate, bivariate or multivariate mixed-effects-meta-regressions executed with a 1000-boastrapping^33,34^. The meta-variables in the regressions were inputted as continuous variables (N and Milkprod2020, WB income, HDI2021, Milkprod2020, and SuDI) or discrete/categorical variable (e.g., PMF type, nation, detection method, and continent). The outcome variables in the meta-regression models were the regression intercepts/coefficients, the coefficients of determinants (R^2^), and associated test of explanatory variable moderating effects or influences.

### Software

All computations in section “Statistical analysis” were performed in R v.4.3.0 (2023-04-21 ucrt) with functions enriched by metafor v.3.8-1, PerformanceAnalytics v.2.0.4, meta v.6.1-0 and dmetar v.0.0.9000 packages^33,35–37^.

## Results

### General description

A total of 3761 records were found from direct database search upon aggregation of which 3187 documents were duplicates (Figure S1). We screened 574 abstracts and titles, 80 of which were eligible for inclusion were retrieved for data extraction. We excluded 12 articles for various reasons including wrong samples and non-availability of full text/access. The final data was extracted from 68 studies which were disaggregated into 88 sub-studies in the models according to sample varieties reported in the studies.

### Data features and regional distribution

**Figure 1:**
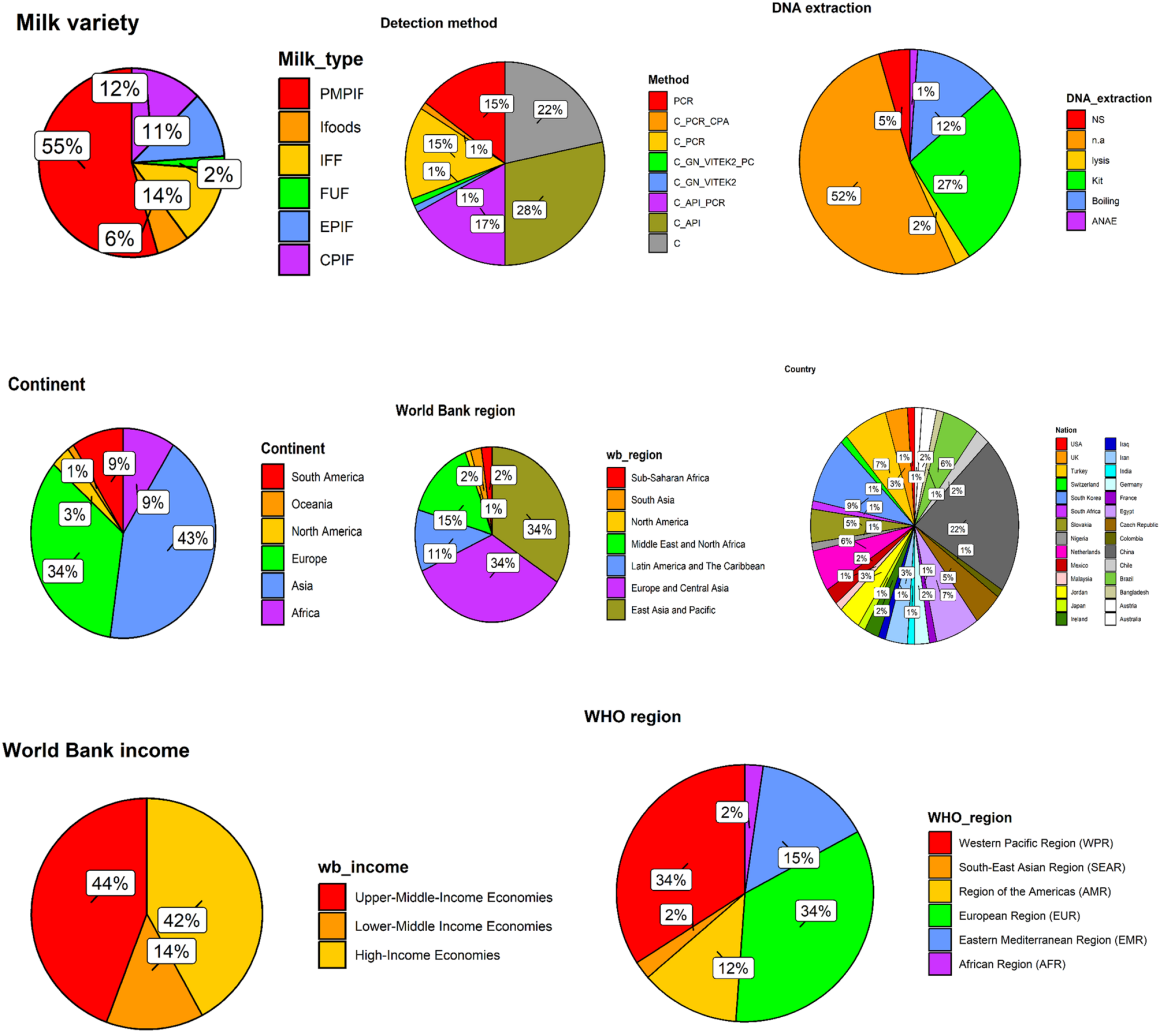
Descriptive summary of the studies on Cz prevalence in PMF.

Figure 1 and Table S1 present the descriptive characteristic of the disaggregated *Cz*-PMF studies. An average of 19.35 ± 33.97 and 297.07 ± 716.09 *Cz*-positivity and sample size (N) records (Table S1) were found in 88 disaggregated studies (details in Table 1). The sample varieties included PIF(48/88, 55.0%), IFF (12/88, 14.0%), CPIF (11/88, 13.0%), EPIF (10/88, 11.0%), Ifoods (5/88, 5.7%), and FUF (2/88; 2.3%). Culture (C) (19/88, 22.0%)), C and API (25/88, 28.0%), C, API, and PCR (15/88, 17.0%), PCR (13/88; 15.0%), and were the most common detection for Cz. Where DNA extraction was required, the use of kits (24/88; 27.0%) was prevalent than the boiling (11/88; 13.0%), lysis(2/88; 2.3%), and automated nucleic acid extraction (ANAE) (1/88;1.1%). The contribution from different countries varies from 1/88 (1.1%) (Australia, Bangladesh, Colombia, France, India, Iraq, Japan, Mexico, Netherlands, Nigeria, South Africa, Switzerland, and USA), 2/88 (2.3%) (Austria, Chile, Germany, Mexico, and Ireland,), 3/88 (3.4%) Jordan, UK, and Iran), 4/88 (4.5%) (Czech Republic, Slovakia), 5/88 (5.7%) (Netherlands), 6/88 (6.8%) (Egypt, Turkey), 8/88 (9.1%) (South Korea) with the highest from China (19/88; 22.0%). The continent of Asia (38/88; 43.0%) had the highest studies of Cz, followed by Europe (30/88; 34.0%), Africa and South America (8/88; 9.1%), North America (3/88; 3.4%), and Oceania (1/88; 1.1%). Both regions of East Asia and Pacific and Europe and Central Asia (30/88 (34.0% each) had the highest *Cz*-PMF studies, followed by Middle East and North Africa (13/88; 15.0%), Latin America and The Caribbean (10/88; 11.0%), South Asia and Sub-Saharan Africa (2/88; 2.3%% each), and North America(1/88; 1.1%) among the world bank classification. Among the world bank income grouping, *Cz*-PMF studies declined from Upper-Middle-Income Economies (UMIE: 39/88; 44%), High-Income Economies (HIE: 37/88; 42%) to Lower-Middle Income Economies (LMIE: 12/88; 14%). Cz-studies also reduced from 30/88 (34%)in the European Region (EUR) and Western Pacific Region (WPR) to 13/88 (15%)in Eastern Mediterranean Region (EMR), 11/88 (13%) in the Region of the Americas (AMR), and 2/88 (2.3%) in South-East Asian Region (SEAR) and African Region (AFR) each among the WHO regions. According to the HDI, Cz studies distributed as 44/88 (50%) in very high HDI, 40/88 (45%) in high HDI, and 4/88 (4.5%) in the medium HDI region.

**Table 1:** Summary of the disaggregated data included.

SN	Author	P	N	Milk type	Method	DNA extraction	Nation	Continent	World bank region	World bank income	WHO region	HDI group	Milkprod2020	SuDI	HDI2021
1	Badawy et al.^38^	0	120	PIF	C, API, PCR	Kit	Egypt	Africa	MENA	LMIEs	EMR	high HDI	5,089,495	0.752	0.731
2	Y. Li et al.^39^	11	660	PIF	C	n.a	China	Asia	EAP	UMIEs	WPR	high HDI	38,769,118	0.461	0.768
3	Y. Li et al.^39^	41	248	CPIF	C	n.a	China	Asia	EAP	UMIEs	WPR	high HDI	38,769,118	0.461	0.768
4	Liang et al.^40^	32	268	IFF	PCR	n.a	China	Asia	EAP	UMIEs	WPR	high HDI	38,769,118	0.461	0.768
5	Ziver et al.^41^	0	265	CPIF	C	n.a	Turkey	Europe	ECA	UMIEs	EUR	very high HDI	21,839,351	0.703	0.838
6	Costa et al.^42^	20	45	CPIF	PCR	Kit	Brazil	South America	LATC	UMIEs	AMR	high HDI	36,806,788	0.747	0.754
7	Amer et al.^43^	9	80	PIF	C	n.a	Egypt	Africa	MENA	LMIEs	EMR	high HDI	5,089,495	0.752	0.731
8	Tayeb et al.^44^	4	130	PIF	C, GN VITEK2, PCR	Kit	Iraq	Asia	MENA	UMIEs	EMR	medium HDI	404,246	0.681	0.686
9	Hayman et al.^45^	253	5671	EPIF	C, API	n.a	USA	North America	North America	HIEs	AMR	very high HDI	101,276,991	0.163	0.921
10	Mashoufi et al.^46^	5	100	PIF	PCR	Kit	Iran	Asia	MENA	LMIEs	EMR	high HDI	8,364,026	0.602	0.774
11	Mashoufi et al.^46^	8	100	Ifoods	PCR	Kit	Iran	Asia	MENA	LMIEs	EMR	high HDI	8,364,026	0.602	0.774
12	Peng et al.^47^	2	100	PIF	C, API	n.a	China	Asia	EAP	UMIEs	WPR	high HDI	38,769,118	0.461	0.768
13	Demirci et al.^48^	10	100	PIF	C, API	n.a	Turkey	Europe	ECA	UMIEs	EUR	very high HDI	21,839,351	0.703	0.838
14	Demirci et al.^48^	3	20	EPIF	C, API	n.a	Turkey	Europe	ECA	UMIEs	EUR	very high HDI	21,839,351	0.703	0.838
15	Tutar et al.^49^	25	25	PIF	PCR	Kit	Turkey	Europe	ECA	UMIEs	EUR	very high HDI	21,839,351	0.703	0.838
16	Morato-Rodriguez et al.^50^	35	102	IFF	PCR	NS	Colombia	South America	LATC	UMIEs	AMR	high HDI	7,071,404	0.801	0.752
17	Zhang et al.^51^	42	1032	PIF	C, GN VITEK2	n.a	China	Asia	EAP	UMIEs	WPR	high HDI	38,769,118	0.461	0.768
18	Brandao et al.^52^	20	30	IFF	PCR	Kit	Brazil	South America	LATC	UMIEs	AMR	high HDI	36,806,788	0.747	0.754
19	Brandao et al.^52^	7	30	CPIF	PCR	Kit	Brazil	South America	LATC	UMIEs	AMR	high HDI	36,806,788	0.747	0.754
20	Mardaneh and Soltan^53^	9	125	PIF	C, API, PCR	Boiling	Iran	Asia	MENA	LMIEs	EMR	high HDI	8,364,026	0.602	0.774
21	Kakatkar et al.^54^	0	20	PIF	PCR	Boiling	India	Asia	South Asia	LMIEs	SEAR	medium HDI	183,955,490	0.696	0.633
22	Pei et al.^55^	25	2282	PIF	C	n.a	China	Asia	EAP	UMIEs	WPR	high HDI	38,769,118	0.461	0.768
23	Z. Li et al.^56^	119	705	PIF	C, API	n.a	China	Asia	EAP	UMIEs	WPR	high HDI	38,769,118	0.461	0.768
24	Aksu et al.^57^	20	101	IFF	C, PCR	Kit	Turkey	Europe	ECA	UMIEs	EUR	very high HDI	21,839,351	0.703	0.838
25	Parra-Flores et al.^58^	6	128	PIF	C, API	n.a	Chile	South America	LATC	HIEs	AMR	very high HDI	2,283,509	0.678	0.858
26	Fang et al.^59^	67	632	EPIF	C, API	n.a	China	Asia	EAP	UMIEs	WPR	high HDI	38,769,118	0.461	0.768
27	Huang et al.^60^	76	1012	IFF	PCR	Kit	China	Asia	EAP	UMIEs	WPR	high HDI	38,769,118	0.461	0.768
28	Pan et al.^61^	49	399	PIF	C, API	n.a	China	Asia	EAP	UMIEs	WPR	high HDI	38,769,118	0.461	0.768
29	Xu et al.^62^	23	530	PIF	C, API, PCR	n.a	China	Asia	EAP	UMIEs	WPR	high HDI	38,769,118	0.461	0.768
30	Mozrova^63^ et al	0	11	PIF	C, API, PCR	Kit	Czech Republic	Europe	ECA	HIEs	EUR	very high HDI	3,282,371	0.399	2.889
31	Mozrova et al.^63^	0	15	IFF	C, API, PCR	Kit	Czech Republic	Europe	ECA	HIEs	EUR	very high HDI	3,282,371	0.399	3.889
32	Gicova et al.^64^	2	398	PIF	C, API, PCR	Boiling	Slovakia	Europe	ECA	HIEs	EUR	very high HDI	929,540	0.285	0.848
33	Gicova et al.^64^	6	518	CPIF	C, API, PCR	Boiling	Slovakia	Europe	ECA	HIEs	EUR	very high HDI	929,540	0.285	1.848
34	Siqueira-Santos et al.^65^	12	67	PIF	C	n.a	Brazil	South America	LATC	UMIEs	AMR	high HDI	36,806,788	0.747	0.754
35	Siqueira-Santos et al.^65^	0	32	EPIF	C	n.a	Brazil	South America	LATC	UMIEs	AMR	high HDI	36,806,788	0.747	0.754
36	Hochel et al.^66^	2	60	PIF	C, API	n.a	Czech Republic	Europe	ECA	HIEs	EUR	very high HDI	3,282,371	0.399	1.889
37	Hochel et al.^66^	6	54	IFF	C, API	n.a	Czech Republic	Europe	ECA	HIEs	EUR	very high HDI	3,282,371	0.399	0.889
38	Jongenburger et al.^67^	8	2290	PIF	C	n.a	Netherlands	Europe	ECA	HIEs	EUR	very high HDI	14,932,000	0.282	0.941
39	Oonaka et al.^68^	9	149	PIF	C	n.a	Japan	Asia	EAP	HIEs	WPR	very high HDI	7,440,965	0.31	0.925
40	Park et al.^69^	7	102	PIF	PCR	Kit	South Korea	Asia	EAP	HIEs	WPR	very high HDI	1,806,012	0.251	0.925
41	Park et al.^69^	41	86	IFF	PCR	Kit	South Korea	Asia	EAP	HIEs	WPR	very high HDI	1,806,012	0.251	0.925
42	Reich et al.^70^	66	467	PIF	C	n.a	Germany	Europe	ECA	HIEs	EUR	very high HDI	33,188,890	0.351	0.942
43	Reich et al.^70^	4	400	EPIF	C	n.a	Germany	Europe	ECA	HIEs	EUR	very high HDI	33,188,890	0.351	0.942
44	Hoque et al.^71^	1	32	PIF	PCR	Kit	Bangladesh	Asia	South Asia	LMIEs	SEAR	medium HDI	3,578,373	0.681	0.661
45	Ye et al.^72^	10	243	Ifoods	C, PCR	n.a	China	Asia	EAP	UMIEs	WPR	high HDI	38,769,118	0.461	0.768
46	Chap et al.^73^	1	136	FUF	C	n.a	UK	Europe	ECA	HIEs	EUR	very high HDI	15,558,000	0.42	0.929
47	Chap et al.^73^	22	179	Ifoods	C	n.a	UK	Europe	ECA	HIEs	EUR	very high HDI	15,558,000	0.42	0.929
48	OBrien et al.^74^	0	390	PIF	C, API	n.a	Ireland	Europe	ECA	HIEs	EUR	very high HDI	8,561,470	0.35	0.945
49	OBrien et al.^74^	2	80	IFF	C, API	n.a	Ireland	Europe	ECA	HIEs	EUR	very high HDI	8,561,470	0.35	0.945
50	Hein et al.^75^	79	1932	PIF	C, API, PCR	Kit	Austria	Europe	ECA	HIEs	EUR	very high HDI	3,852,260	0.239	1.916
51	Hein et al.^75^	54	136	EPIF	C, API, PCR	Kit	Austria	Europe	ECA	HIEs	EUR	very high HDI	3,852,260	0.239	0.916
52	El-Sharoud et al.^76^	6	112	PIF	C, API, PCR	NS	Egypt	Africa	MENA	LMIEs	EMR	high HDI	5,089,495	0.752	0.731
53	Jaradat et al.^77^	1	69	PIF	C, API, PCR	Kit	Jordan	Asia	MENA	UMIEs	EMR	high HDI	427,948	0.763	0.72
54	Derzelle et al.^78^	23	38	EPIF	C, PCR	ANAE	France	Europe	ECA	HIEs	EUR	very high HDI	26,152,110	0.49	0.903
55	Torres-Chavolla et al.^79^	31	50	PIF	C, API	n.a	Mexico	North America	LATC	UMIEs	AMR	high HDI	12,783,734	0.774	0.754
56	Kaclikova and Turcovsky^80^	3	30	PIF	C, PCR	Kit	Slovakia	Europe	ECA	HIEs	EUR	very high HDI	929,540	0.285	1.848
57	Kaclikova and Turcovsky^80^	1	15	Ifoods	C, PCR	Kit	Slovakia	Europe	ECA	HIEs	EUR	very high HDI	929,540	0.285	1.848
58	Kandhai et al.^81^	18	152	EPIF	C, API	n.a	Netherlands	Europe	ECA	HIEs	EUR	very high HDI	14,932,000	0.282	0.941
59	Gutierrez-Rojo and Torres-Chavolla^82^	39	50	PIF	C, PCR	lysis	Mexico	North America	LATC	UMIEs	AMR	high HDI	12,783,734	0.774	0.754
60	Guillaume-Gentil et al.^83^	77	192	EPIF	C, API	n.a	Netherlands	Europe	ECA	HIEs	EUR	very high HDI	14,932,000	0.282	0.941
61	Shaker et al.^84^	1	18	CPIF	C, API	n.a	Jordan	Asia	MENA	UMIEs	EMR	high HDI	427,948	0.763	0.72
62	Shaker et al.^84^	2	15	IFF	C, API	n.a	Jordan	Asia	MENA	UMIEs	EMR	high HDI	427,948	0.763	0.72
63	Kandhai et al.^85^	16	575	PIF	C, PCR	Kit	Netherlands	Europe	ECA	HIEs	EUR	very high HDI	14,932,000	0.282	0.941
64	Kandhai et al.^85^	1	182	IFF	C, PCR	Kit	Netherlands	Europe	ECA	HIEs	EUR	very high HDI	14,932,000	0.282	0.941
65	Lee et al.^86^	14	95	Ifoods	C, PCR	Kit	South Korea	Asia	EAP	HIEs	WPR	very high HDI	1,806,012	0.251	0.925
66	Zhou et al.^87^	7	13	PIF	C, PCR	NS	China	Asia	EAP	UMIEs	WPR	high HDI	38,769,118	0.461	0.768
67	Craven^88^ et al. 2010	73	253	EPIF	C, API	n.a	Australia	Oceania	EAP	HIEs	WPR	very high HDI	8,858,135	0.156	0.951
68	Sani and Yi^89^	0	74	PIF	C, API	n.a	Malaysia	Asia	EAP	UMIEs	WPR	very high HDI	49,364.52	0.491	0.803
69	Choi et al.^90^	13	58	CPIF	C, API	Boiling	South Korea	Asia	EAP	HIEs	WPR	very high HDI	1,806,012	0.251	0.925
70	Choi et al.^90^	1	13	PIF	C, API	Boiling	South Korea	Asia	EAP	HIEs	WPR	very high HDI	1,806,012	0.251	0.925
71	Ragab et al.^91^	24	50	PIF	C, PCR	Boiling	Egypt	Africa	MENA	LMIEs	EMR	high HDI	5,089,495	0.752	0.731
72	Lehner et al.^92^	10	170	PIF	C, PCR	NS	Switzerland	Europe	ECA	HIEs	EUR	very high HDI	3,840,200	0.26	0.962
73	El-Gamal et al.^93^	12	90	PIF	C	n.a	Egypt	Africa	MENA	LMIEs	EMR	high HDI	5,089,495	0.752	0.731
74	Witthuhn et al.^94^	4	22	PIF	C, PCR	lysis	South Africa	Africa	SSA	UMIEs	AFR	high HDI	3,837,000	0.678	0.713
75	Iversen and Forsythe^95^	5	154	PIF	C	n.a	UK	Europe	ECA	HIEs	EUR	very high HDI	15,558,000	0.42	0.929
76	Aigbekaen and Oshoma,^96^	20	70	PIF	C	n.a	Nigeria	Africa	SSA	LMIEs	AFR	medium HDI	531,586.8	0.581	0.534
77	Li et al.^97^	12	85	CPIF	C	n.a	China	Asia	EAP	UMIEs	WPR	high HDI	38,769,118	0.461	0.768
78	Li et al.^97^	0	33	PIF	C	n.a	China	Asia	EAP	UMIEs	WPR	high HDI	38,769,118	0.461	0.768
79	Li et al.^97^	0	15	IFF	C	n.a	China	Asia	EAP	UMIEs	WPR	high HDI	38,769,118	0.461	0.768
80	Choi et al.^98^	3	23	CPIF	C, API, PCR	Kit	South Korea	Asia	EAP	HIEs	WPR	very high HDI	1,806,012	0.251	0.925
81	Lou et al.^99^	2	16	CPIF	C, API, PCR	Boiling	China	Asia	EAP	UMIEs	WPR	high HDI	38,769,118	0.461	0.768
82	Lou et al.^99^	1	59	PIF	C, API, PCR	Boiling	China	Asia	EAP	UMIEs	WPR	high HDI	38,769,118	0.461	0.768
83	Gokmen et al.^100^	3	110	PIF	C, API	n.a	Turkey	Europe	ECA	UMIEs	EUR	very high HDI	21,839,351	0.703	0.838
84	Kim et al.^101^	17	36	CPIF	C, API, PCR	Boiling	South Korea	Asia	EAP	HIEs	WPR	very high HDI	1,806,012	0.251	0.925
85	Jung and Park^102^	3	25	PIF	C, API	n.a	South Korea	Asia	EAP	HIEs	WPR	very high HDI	1,806,012	0.251	0.925
86	Zhao et al.^103^	3	236	PIF	C, PCR, CPA	Boiling	China	Asia	EAP	UMIEs	WPR	high HDI	38,769,118	0.461	0.768
87	Parra et al.^104^	2	72	PIF	C, API	n.a	Chile	South America	LATC	HIEs	AMR	very high HDI	2,283,509	0.678	0.858
88	El-Sharoud et al.^105^	2	35	FUF	C, API	n.a	Egypt	Africa	MENA	LMIEs	EMR	high HDI	5,089,495	0.752	0.731

### Cz positivity and relationship with essential variables

**Figure 2:**
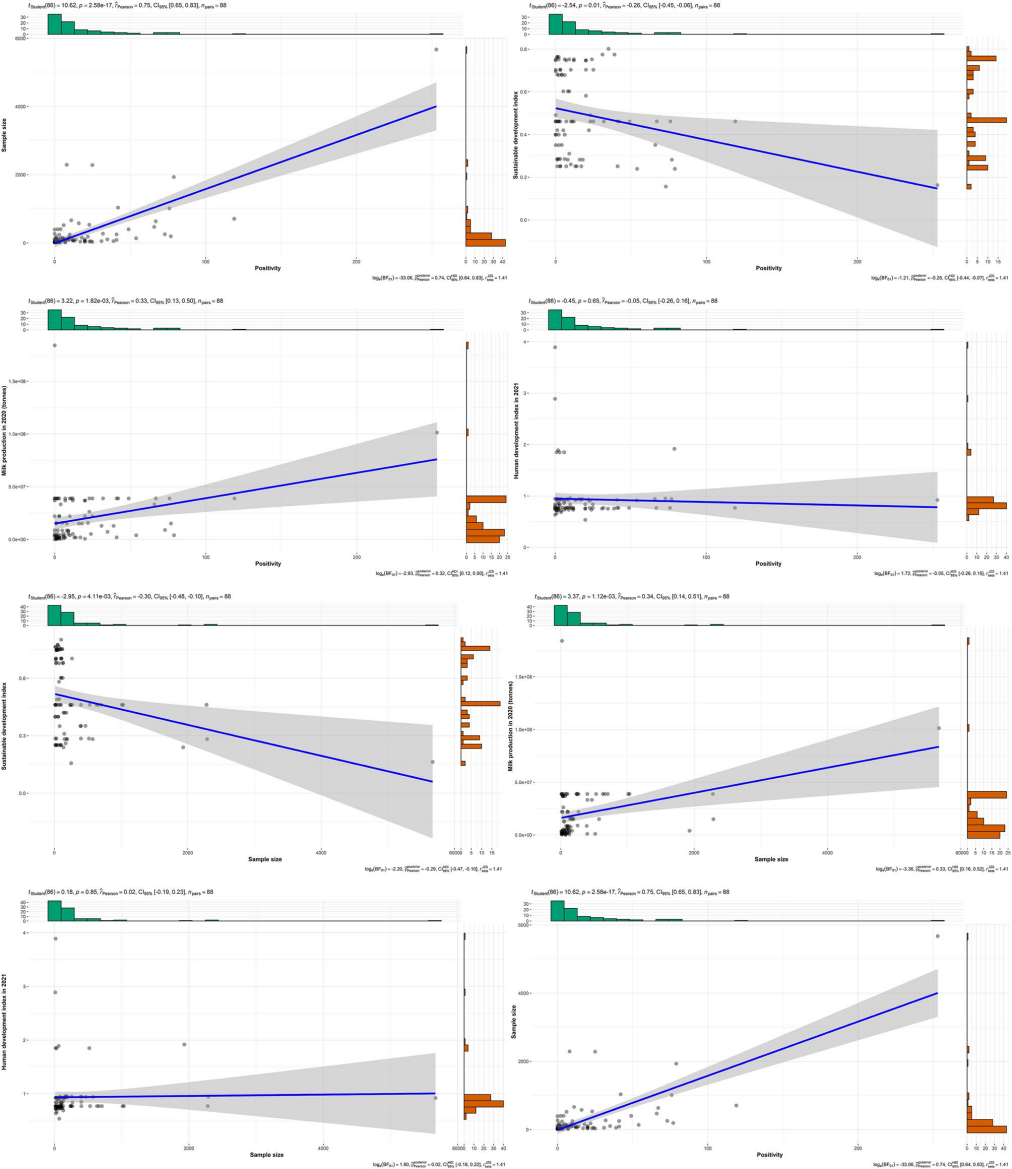
Bivariate characteristics among Cz positivity records, sample size and region-specific data.

The bivariate associations among Cz positivity records, sample size and region-specific data (Milkprod2020, WB income, HDI2021, Milkprod2020, and SuDI) is presented in Fig. 2. There was a strong positive correlation between sample size and Cz positivity record (r = 0.75, *p* = 2.58–17), Milkprod2020 weakly correlated with Cz positivity (r = 0.33, *p* = 1.82e−03) as well as Milkprod2020 weakly correlated with sample size (r = 0.34, *p* = 1.12e−3.01). However, weak correlation exists between HDI2021 and sample size (r = 0.02, *p* = 0.85), SuDI inversely and moderately correlated with sample size (r = − 0.30 *p* = 4.11e−3), SuDI also inversely and moderately correlated with Cz positivity records.

### Global, method- and sample-based prevalence of Cz in PMF

**Figure 3:**
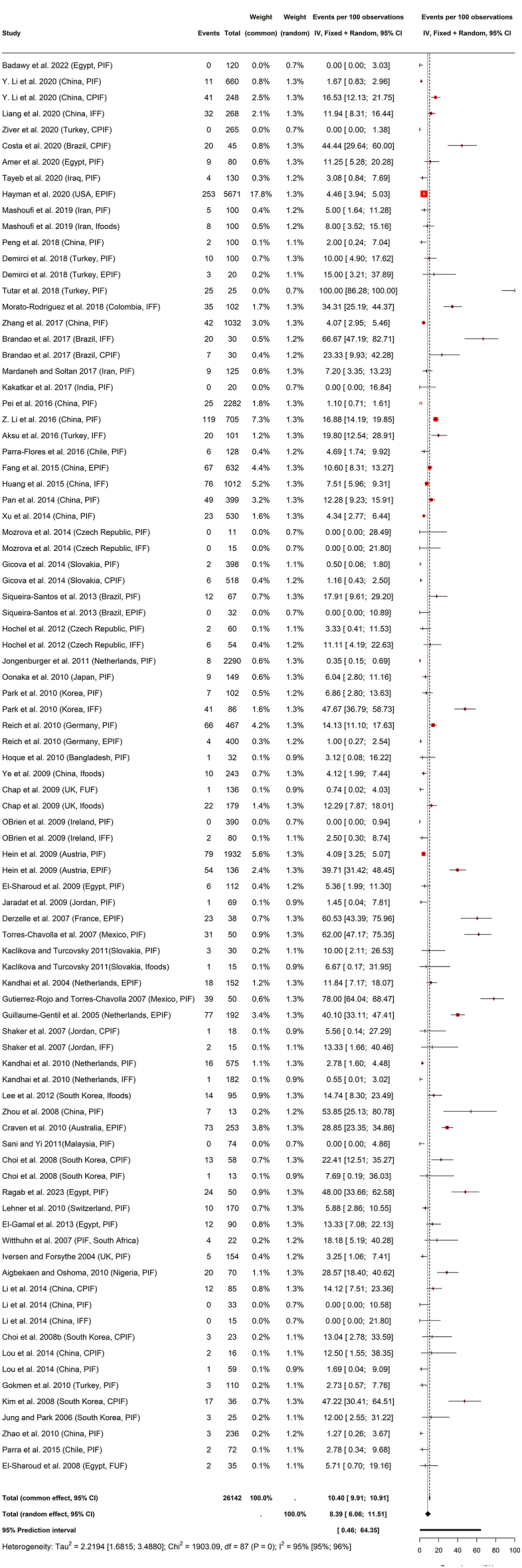
Global prevalence of Cz in PMF.

Figure 3 presents the global Cz prevalence in PMF. The global prevalence of Cz in PMF was 8.39% (95%CI 6.06–11.51, PI: 0.46–64.35; I^2^ = 95%, 95%CI 95–96), which upon LOSOCV resulted to 7.66% (6.39; 9.15; PI: 3.10–17.70; I^2^ = 61%, *p* < 0.01) (Figure S2). The Eggers' test of funnel plot asymmetry for the global prevalence (intercept = − 0.1, 95% CI − 1.66–1.46, t = − 0.126, *p* = 0.90) as well as its trim-fill results did not indicate presence of small-study effects or bias (supplementary material).

The prevalence of CZ in PMF varies significantly (Test for method differences: *p* < 0.01) according to detection method with the highest prevalence of 20.20% (7.74–43.31; I^2^ = 95, 93–96) achieved by PCR, followed by 16.13% (6.14–36.10; I^2^ = 95%, 93–97) obtained by combination of C and PCR, 9.09% (5.49–14.68; I^2^ = 96%, 95–97) obtained by C and API. The prevalence of Cz in the samples was less than the global prevalence when C and GN_VITEK2 (4.07%, 2.95–5.46), C, GN_VITEK2 and PCR (34.07%, 2.95–5.46), C (4.53%, 2.21–9.04; I^2^ = 95%, 93–96), and C, API, and PCR (5.16%, 2.23–11.50; I^2^ = 95%, 93–96) were used (Table 2).

**Table 2:** Subgroup, LOSOCV, and regional prevalence of PMF.

Subgroup model: crude statistics	Prevalence	95%CI	I^2^	95%CI	Cochrane Q test
DNA extraction**					
Kit: 406/4996, k = 24, 8.13%	10.69	5.25–20.48	95	94–96	${\chi }_{23}^{2}$= 466.49 (*p* < 0.01)
Boiling: 101/1567, k = 12, 6.45%	6.55	2.01–19.30	94	91–96	${\chi }_{10}^{2}$= 162.71 (*p* < 0.01)
Automated nucleic acid extraction	60.53	43.39–75.96	NA	NA	NA
Lysis: 43/72, k = 2, 59.72%	47.77	0.00–100	94	83–98	${\chi }_{1}^{2}$= 18.17(*p* < 0.01)
NA: 1095/19,110, k = 46, 5.73%	6.39	4.33–9.35	96	95–96	${\chi }_{45}^{2}$= 1008.79 (*p* < 0.01)
NR: 58/397, k = 4, 14.61%	17.45	1.88–69.98	94	88–97	${\chi }_{1}^{2}$=22.49 (*p* < 0.01)
Continent**					
Africa: 77/579, k = 8, 13.3%	13.00	4.58–31.74	87	77–93	${\chi }_{7}^{2}$= 55.29 (*p* = 0.34)
Asia: 661/9838, k = 38, 6.72%	7.59	5.24–10.87	93	91–94	${\chi }_{37}^{2}$= 507.02 (*p* < 0.01)
Oceania: 73/253, k = 1, 28.85%	28.85	23.35–34.86	NA	NA	NA
Europe: 467/9195, k = 30, 5.08%	5.45	2.66–10.86	96	95–97	${\chi }_{29}^{2}$= 674.9 (*p* < 0.01)
North America: 323/5771, k = 3, 5.6%	38.77	0.20–99.50	99	99–100	${\chi }_{2}^{2}$= 285.79 (*p* < 0.01)
South America: 175/759, k = 9, 23.06%	18.12	5.40–46.15	90	98–94	${\chi }_{7}^{2}$= 72.12 (*p* < 0.01)
HDI					
High HDI: 769/9910, k = 40, 7.76%	10.33	6.61–15.79	95	94–96	${\chi }_{39}^{2}$= 751.25 (*p* < 0.01)
Medium HDI: 25/252, k = 4, 9.92%	6.57	0.74–39.84	88	72–95	${\chi }_{3}^{2}$= 25.38 (*p* = 1.00)
Very high HDI: 909/15,980, k = 44, 5.69%	7.03	4.19–11.55	96	95–97	${\chi }_{43}^{2}$= 1103.85 (*p* < 0.01)
Method**					
C: 257/7702, k = 19, 3.34%	4.53	2.21–9.04	95	93–96	${\chi }_{18}^{2}$= 357.36 (*p* < 0.01)
C, API: 745/9406, k = 25, 7.92%	9.09	5.49–14.68	96	95–97	${\chi }_{24}^{2}$= 590.06 (*p* < 0.01)
C, API & PCR: 203/4100, k = 15, 4.95%	5.16	2.23–11.50	95	93–96	${\chi }_{14}^{2}$= 283.13 (*p* < 0.01)
C, GN VITEK2: 42/1032, k = 1, 4.07%	4.07	2.95–5.46	NA	NA	NA
C, GNVITEK2 & PCR: 4/130, k = 1, 3.08%	3.08	0.84–7.69	NA	NA	NA
C & PCR: 172/1584, k = 13, 10.86%	16.13	6.14–36.10	95	93–97	${\chi }_{12}^{2}$= 257.21 (*p* < 0.01)
C, PCR & CPA: 3/236, k = 1, 1.27%	1.27	0.26–3.67	NA	NA	NA
PCR: 277/1952, k = 13, 14.19%	20.20	7.74–43.31	95	93–96	${\chi }_{12}^{2}$= 229.88 (*p* < 0.01)
Milk type*					
CPIF: 122/1342, k = 11, 9.09%	12.73	4.64–30.40	90	85–95	${\chi }_{10}^{2}$= 103.36 (*p* < 0.01)
EPIF: 572/7526, k = 10, 7.6%	14.53	5.14–34.80	99	98–99	${\chi }_{9}^{2}$= 608.33 (*p* < 0.01)
FUF: 3/171, k = 2, 1.75%	2.32	0.00–99.99	65	0–92	${\chi }_{1}^{2}$= 2.87 (*p* = 0.09)
IFF: 235/1960, k = 12, 11.99%	12.84	5.02–29.09	94	92–96	${\chi }_{11}^{2}$= 192.52 (*p* < 0.01)
Ifoods: 55/632, k = 5, 8.7%	8.92	4.52–16.85	78	18–88	${\chi }_{4}^{2}$= 12.62 (*p* = 0.01)
PIF: 716/14,511, k = 48, 4.9%	6.25	3.88–9.92	95	94–95	${\chi }_{47}^{2}$= 866.46 (*p* < 0.01)
WHO**					
African Region (AFR): 24/92, K = 2, 26.09%	26.39	1.92–86.79	0	–	${\chi }_{1}^{2}$= 0.9 (*p* = 0.34)
Eastern Mediterranean Region (EMR): 83/1044, k = 13, 7.95%	7.62	3.95–14.17	85	76–91	${\chi }_{12}^{2}$= 879.48 (*p* < 0.01)
European Region (EUR): 467/9195, k = 30, 5.08%	5.45	2.66–10.86	96	95–97	${\chi }_{29}^{2}$= 674.9 (*p* < 0.01)
Region of the Americas (AMR): 425/6277, k = 11, 6.77%	22.61	8.02–49.46	98	98–99	${\chi }_{10}^{2}$= 528.88 (*p* < 0.01)
South–East Asian Region (SEAR): 1/52, k = 2, 1.92%	2.85	0.55–13.58	0	–	${\chi }_{1}^{2}$= 0.03 (*p* = 0.87)
Western Pacific Region (WPR): 703/9482, k = 30, 7.41%	8.81	5.62–13.56	95	94–96	${\chi }_{29}^{2}$= 568.92 (*p* < 0.01)
Income*					
High-Income Economies: 848/15,285, k = 37, 5.55%	6.78	4.08–11.07	97	96–97	${\chi }_{36}^{2}$= 1055 (*p* < 0.01)
Lower–Middle Income Economies: 96/934, k = 12, 10.28%	9.21	4.47–18.04	87	78–92	${\chi }_{11}^{2}$= 82.21 (*p* = 0.77)
Upper–Middle-Income Economies: 759/9923, k = 39, 7.65%	8.41	5.92–16.92	95	94–96	${\chi }_{38}^{2}$= 561.13 (*p* < 0.01)
World bank region**					
EAP: 703/9482, k = 30, 7.41%	8.81	5.62–13.56	95	94–96	${\chi }_{29}^{2}$= 568.92 (*p* < 0.01)
ECA: 467/9195, k = 30, 5.08%	5.45	2.66–10.86	96	95–97	${\chi }_{29}^{2}$= 674.9 (*p* < 0.01)
LATC: 172/606, k = 10, 28.38%	26.46	9.16–56.20	93	89–95	${\chi }_{9}^{2}$= 122.86 (*p* < 0.01)
MENA: 83/1044, k = 13, 7.95%	7.62	3.95–14.17	85	76–91	${\chi }_{12}^{2}$= 79.48(*p* = 0.27)
North America: 253/5671, k = 1, 4.46%	4.46	3.94–5.03	NA	NA	
South Asia: 1/52, k = 2, 1.92%	2.85	0.55–13.58	0%	–	${\chi }_{1}^{2}$= 0.03 (*p* = 0.87)
Sub-Saharan Africa: 24/92, k = 2, 26.09%	26.39	1.92–86.79	0%	–	${\chi }_{1}^{2}$= 0.92 (*p* = 0.34)

K, number of studies pooled together; *Test for subgroup differences: *p* > 0.05; **Test for subgroup differences: *p* < 0.01.

The prevalence of Cz in PMF was not considerably different (test for subgroup differences: *p* > 0.05) with the highest recorded in EPIF (14.53%, 5.14–34.80; I^2^ = 99%, 98–99), followed by IFF (12.84%, 5.02–29.09; I^2^ = 94%, 92–96), CPIF (12.73%, 4.64–30.40, I^2^ = 90%, 85–95), Ifoods (8.92%, 4.52–16.85; I^2^ = 78%, 18–88), PIF (6.25%, 3.88–9.92; I^2^ = 95%, 94–95), and FUF (2.32%, 0.00–99.99; I^2^ = 65%, 0–92) (Table 2). The prevalence of Cz in PMF was significantly different with DNA extraction method (test for DNA extraction differences: *p* < 0.01) with the use of kit resulted to higher prevalence (10.69%, 5.25–20.48, I^2^ = 95%, 94–96) compared with boiling method (6.55%, 2.01–19.30, I^2^ = 94%, 91–96) (Table 2).

### Regional prevalence of Cz in PMF

Also, Cz prevalence in PMF was significantly difference across continents (Test for continent differences: *p* < 0.01) with highest prevalence was recorded in North America (38.77%, 0.20–99.50, I^2^ = 99%, 99–100), followed by South America (18.12%, 5.40–46.15, I^2^ = 90%, 98–94), Africa (13.00%, 4.58–31.74, I^2^ = 87%, 77–93), Asia (7.59%, 5.24–10.87, I^2^ = 93%, 91–94), and Europe (5.45%, 2.66–10.86, I^2^ = 96%, 95–97) (Table 2). An individual study from Oceania (28.85%, 23.35–34.86) recorded high prevalence of Cz in PMF.

Cz prevalence in PMF differs across HDI (F.gure 4; Table 2). Cz prevalence in PMF was 10.33% (6.61–15.79, I^2^ = 95%, 94–96), 7.03% (4.19–11.55, I^2^ = 96%, 95–97), and 6.57% (0.74–39.84, I^2^ = 88%, 72–95) in high HDI, very high HDI, and medium HDI, respectively. However, the HDI differences of prevalence was insignificantly different (*p* = 0.47).

For the WHO, Cz prevalence in PMF was 26.39% (1.92–86.79, I^2^ = 0) in AFR, 22.61% (8.02–49.46; I^2^ = 98%, 98–99) in AMR, 8.81% (5.62–13.56, I^2^ = 95%, 94–96) in WPR, 7.62% (3.95–14.17, I^2^ = 85%, 76–91) in EMR, 5.45% (2.66–10.86, I^2^ = 96%, 95–97) in EUR, 2.85% (0.55–13.58, I^2^ = 0%) in SEAR and was significantly different across the regions (test for WHO region differences: *p* < 0.01). However, the Cz prevalence in PMF was not significantly difference (Test for world income region differences: *p* = 0.50) among world bank income region with the highest recorded in Lower–Middle Income Economies (9.21%, 4.47–18.04, I^2^ = 87%, 78–92), Upper–Middle–Income Economies (8.41%, 5.92–16.92, I^2^ = 95%, 94–96), and High-Income Economies (6.78%, 4.08–11.07, I^2^ = 97%, 96–97) (Table 2).

The world bank regional classification reevealed a significant different in the prevalence of Cz in PMF (test for world bank regional differences: *p* = 0.01) with LATC having the highest valued at 26.46% (9.16–56.20, I^2^ = 93%, 89–95), then EAP with 8.81% (5.62–13.56, 95%, 94–96), MENA (7.62%, 3.95–14.17, I^2^ = 85%, 76–91), ECA (5.45%, 2.66–10.86, I^2^ = 96%, 95–97), North America (4.46%, 3.94–5.03), and South Asia (2.85%, 0.55–13.58, I^2^ = 0%)(Table 2).

### Factors moderating Cz prevalence in PMF

Table 3 presents meta-regressions of regional and observational factors influencing *Cz* prevalence in PMF based on 1000-permutations. The models accounted for 0.48 to 70.30% (R^2^) of the true estimates of Cz prevalence in PMF. However, among forty-five metaregression models fitted, the test for the moderators were only significant in 26 models. In a univariate meta-regression model, Nation (F_27,60_ = 1.6691, *p* = 0.04), Method (F_7,80_ = 2.2644, *p* = 0.04), world bank region (F_6,81_ = 2.1455, *p* = 0.05), WHO region (F_5,82_ = 2.1658, *p* = 0.04), and N (F_1,86_ = 5.3564, *p* = 0.02) explained 53.88%, 19.62%, 19.03%, 15.63%, and 9.22% of the true differences in the Cz prevalence in PMF, respectively. Similarly, bivariate meta-regression of N and Nation (R^2^ = 70.26%, F_28,59_ = 2.5541, *p* = 0.002), milk type and Nation (R^2^ = 66.14%, F_32,55_ = 1.8324, *p* = 0.02), N and Continent (R^2^ = 37.43%, F_6,81_ = 4.8212, *p* = 0.001), milk type and world bank region (R^2^ = 34.67, F_11,76_ = 2.1717, *p* = 0.02), milk type and continent (R^2^ = 30.42%, F_10,77_ = 2.1295, *p* = 0.03), N and WHO region (R^2^ = 29.54%, F_6,81_ = 3.5211, *p* = 0.002), milk type and Method (R^2^ = 28.53%, F_12,75_ = 1.8910, *p* = 0.04), N and method (R^2^ = 26.14%, F_8,79_ = 2.5432, *p* = 0.02), N and milk type (R^2^ = 22.61%, F_6,81_ = 2.3695, *p* = 0.02), Milk type and SuDI (R^2^ = 20.65%, F_6,81_ = 2.1574, *p* = 0.05), and DNA extraction and SuDI (R^2^ = 19.11%, F_6,81_ = 2.3604, *p* = 0.03) accounted for the respective percent (R^2^ value) of the actual Cz prevalence estimate in PMF. A tri-variate meta-regression combination of N, nation and HDI2021 (R^2^ = 70.30%, F_3,84_ = 2.4911, *p* = 0.07), N, world bank region and HDI2021 (R^2^ = 35.02%, F_8,79_ = 3.1992, *p* = 0.005), N, method and HDI2021 (R^2^ = 28.68, F_9,78_ = 2.4392, *p* = 0.01), N, milk type and HDI2021 (R^2^ = 25.71%, F_7,80_ = 2.3558, *p* = 0.02), N, SuDI and HDI2021 (R^2^ = 12.52%, F_3,84_ = 2.4911, *p* = 0.05) explained the associated percents (R^2^ values)of the true differences in Cz prevalence estimate in PMF respectively.

**Table 3:** A 1000-permutation based meta-regressions of regional and observational factors influencing *Cz* prevalence in PMF.

Univariate/bivariate/multivariate	β_0_ ± SE	I^2^ (%)	R^2^ (%)	Test of moderators (F_d1,d2_ = Q, p)
N + Nation + HDI2021	− 0.4423 ± 1.2580	87.73	70.30	F_3,84_ = 2.4911, *p* = 0.0680
N + Nation,	− 0.5612 ± 1.0963	87.98	70.26	F_28,59_ = 2.5541, *p* = **0.0020**
Milk type + Nation,	− 1.4163 ± 1.4199	88.47	66.14	F_32,55_ = 1.8324, ***p* = 0.0210**
Nation	− 0.9025 ± 1.2877	92.12	53.88	F_27,60_ = 1.6691, *p* = **0.0400**
N + Continent	− 1.8570 ± 0.5051	94.06	37.43	F_6,81_ = 4.8212, *p* = **0.0010**
N + world bank region + HDI2021	− 1.6877 ± 0.4929	94.26	35.02	F_8,79_ = 3.1992, *p* = **0.0090**
N + world bank region + HDI2021	− 1.6877 ± 0.4929	94.26	35.02	F_8,79_ = 3.1992, *p* = **0.0050**
N + world bank region + HDI2021 + WHO region	− 1.6877 ± 0.4929	94.26	35.02	F_8,79_ = 3.1992, *p* = **0.0020**
Method + world bank region + HDI2021 + WHO region	− 2.6739 ± 0.5598	93.85	34.84	F_14,73_ = 2.0432, *p* = **0.0240**
Milk type + world bank region + HDI2021 + WHO region	− 1.9490 ± 0.6574	93.95	34.82	F_12,75_ = 1.9760, *p* = **0.0360**
Milk type + world bank region,	− 2.0871 ± 0.4813	94.22	34.67	F_11,76_ = 2.1717, *p* = **0.0210**
Milk type + Continent	− 0.8841 ± 0.7646	94.37	30.42	F_10,77_ = 2.1295, *p* = **0.0260**
N + WHO region	− 1.1598 ± 1.0172	94.99	29.54	F_6,81_ = 3.5211, *p* = **0.0020**
N + Method + HDI2021	− 2.3793 ± 0.5168	94.54	28.68	F_9,78_ = 2.4392, *p* = **0.0130**
Milk type + Method	− 2.2934 ± 0.5660	95.11	28.53	F_12,75_ = 1.8910, *p* = **0.0370**
Continent + world bank region + HDI2021 + WHO region	− 1.5091 ± 0.9997	94.69	27.81	F_10,77_ = 1.9519, *p* = **0.0390**
Milk type + WHO region	− 0.5100 ± 1.1835	95.09	26.83	F_10,77_ = 1.8272, *p* = 0.0560
N + Method	− 2.8268 ± 0.3649	94.86	26.14	F_8,79_ = 2.5432, *p* = **0.0200**
N + Milk type + HDI2021	− 1.2650 ± 0.6140	94.83	25.71	F_7,80_ = 2.3558, *p* = **0.0220**
N + Milk type ,	− 1.8303 ± 0.4701	95.26	22.61	F_6,81_ = 2.3695, *p* = **0.0240**
Milk type + SuDI	− 2.9677 ± 0.6423	95.76	20.65	F_6,81_ = 2.1574, *p* = **0.0460**
Milkprod2020 + world bank region + HDI2021 + WHO region	− 1.8742 ± 0.6293	95.25	20.60	F_8,79_ = 1.6798, *p* = 0.1160
Method	− 3.0318 ± 0.3592	95.88	19.62	F_7,80_ = 2.2644, *p* = **0.0420**
DNA extraction + SuDI	− 0.1528 ± 1.5400	96.01	19.11	F_6,81_ = 2.3604, *p* = **0.0340**
world bank region	− 2.3524 ± 0.2832	95.59	19.03	F_6,81_ = 2.1455, *p* = **0.0530**
WHO region	− 1.1932 ± 1.0784	95.99	15.63	F_5,82_ = 2.1658, *p* = **0.0410**
Milk type + world bank income	− 2.22630.5406	95.99	15.01	F_7,80_ = 1.3014, *p* = 0.2680
Milk type + HDI2021	− 1.2986 ± 0.6354	95.97	14.75	F_6,81_ = 1.4755, *p* = 0.1570
Milk type + HDI group	− 1.6694 ± 0.5139	96.02	14.62	F_7,80_ = 1.2629, *p* = 0.2440
N + SuDI + HDI2021	− 2.16110.8037	95.80	12.52	F_3,84_ = 2.4911, *p* = 0.0657
N + SuDI + HDI2021	− 2.1611 ± 0.8037	95.80	12.52	F_3,84_ = 2.4911, *p* = **0.0530**
Milk type + Milkprod2020	− 1.7813 ± 0.5162	95.87	12.29	F_6,81_ = 1.1946, *p* = 0.3176
N + Milkprod2020 + HDI2021	− 1.7469 ± 0.5058	95.84	12.00	F_3,84_ = 2.3306, *p* = 0.0720
N + HDI2021	− 1.7021 ± 0.4388	95.88	11.87	F_2,85_ = 3.5179, *p* = **0.0270**
N + SuDI + Milkprod2020	− 2.7450 ± 0.5231	95.91	11.37	F_3,84_ = 2.2047, *p* = 0.0890
N + SuDI	− 2.7354 ± 0.5194	96.05	11.05	F_2,85_ = 3.2935, *p* = **0.0300**
Milk type	− 1.9218 ± 0.4898	96.29	10.77	F_5,82_ = 1.2873, *p* = 0.2150
N + HDI group	− 2.0197 ± 0.2598	96.01	10.76	F_3,84_ = 2.1626, *p* = 0.0770
N + world bank income	− 2.3765 ± 0.2835	96.02	10.44	F_3,84_ = 2.1192, *p* = 0.0920
N + Milkprod2020	− 2.2739 ± 0.2279	96.01	9.92	F_2,85_ = 2.7911, *p* = 0.0560
N	− 2.2085 ± 0.1867	96.17	9.22	F_1,86_ = 5.3564, *p* = **0.0230**
SuDI	− 3.1543 ± 0.4775	96.66	5.00	F_1,86_ = 2.9954, *p* = 0.0770
HDI group	− 2.1674 ± 0.2593	96.79	2.04	F_2,85_ = 0.6797, *p* = 0.4790
world bank income	− 2.6158 ± 0.2705	96.78	1.94	F_2,85_ = 0.7052, *p* = 0.4880
Milkprod2020	− 2.3128 ± 0.2335	96.56	0.48	F_1,86_ = 0.2480, *p* = 0.6050

The table was sorted in descending order of R^2^; bold *p* values indicated statistical significance.

## Discussion

*Cz* remains a significant threat to newborn’s health in powdered milk and flours. This is being majorly promoted by the inability of conventional food decontamination procedures applied in disinfection of powdered milk and flours to get ridd off Cz in the powdered mik and flours, and their processing facilities. Findings from this study revealed an average sample size of 297.07 ± 716.09 in the studies. This average sample size is consistent with existing international guidance of 30 samples at 10 g as well as the stringent 180 sample sizes at 25 g for *Cronobacter*^106^. Generally, a systematic/stratified random sampling practices with increasing sample size, have been reported to increase detection power of *Cronobacter* in powdered milk^106^. Among the disaggregated studies of powdered milk and flour sample, PIF (55.0%) had the highest representation, followed by IFF (14.0%), CPIF (13.0%), EPIF (11.0%), Ifoods (5.7%), and FUF (2.3%). This further strengthened *Cz* as a significant hazard in PIF. Cz is hard to detect because of heterogenous localization and low-level contamination in PIF^106^. The distribution of the various method used in Cz detection largely favoured traditional cultural method with or without API and less of PCR with or without C and API. *Cz* is hard to detect in powder milk and flour due to focalized low-level contamination as well as its desiccation-tolerance/resistance which may renders Cz culturable after a prolong period^16,106–109^.

The distribution of the studies across regions showed a general low participation in surveillances of Cz in powdered milk and flour. This might be due in part to lack of Cz awareness. Thus, a more action is needed even in the represented countries including Australia, Bangladesh, Colombia, France, India, Iraq, Japan, Mexico, Netherlands, Nigeria, South Africa, Switzerland, USA, Austria, Chile, Germany, Mexico, Ireland, Jordan, UK, Iran, Czech Republic, Slovakia, Netherlands, Egypt, Turkey,, South Korea, and China. Meanwhile, the continent of Asia (43.0%) had the highest studies that focused on Cz in powdered milk and flour, followed by Europe (34.0%), Africa and South America (9.1% each), North America (3.4%), and Oceania (1.1%). It is unknown whether this pattern is associated with consumption or production of powdered milk and flours in the regions. Thus, more action is needed across the regions.

Furthermore, the finding from this study showed that EAP and ECA had the highest *Cz*-PMF studies, followed by MENA, LATC, South Asia and Sub-Saharan Africa, and North America. However, the overall result depicts that irrespective of the region, Cz monitoring in powdered milk and flours are still inadequate. It should be seen as 6a matter of priority to include Cz as one of the priority pathogens for monitoring in PIF especially. Also, the monitoring of Cz in powdered milk and flours received declined attention UMIE (44%) to LMIE (14%) as well as from EUR (24%) to SEAR and AFR (2.3%), and very high HDI (45%) to medium HDI (4.5%). This generally depicts inadequacy in the monitoring and required urgent actions. A region- or super region-specific and aggressive Cz monitoring program in PMF may significantly improve sustainable safety of milk globally.

The need for adequate sample size for detection or assessment of Cz contamination in powdered milk and flour is further strengthened by the observed high and considerable correlational affinity between sample size and Cz positivity in this study (Fig. 2). As such, adequate sample size plays important roles in accurate assessment of Cz contamination. This aligned with previous report^106^. In the same light, the significant positive association of milk production with Cz positivity and sample size provide an insight into increasing contamination with unit increase in milk production and the need for increasing sample size when production increases to ascertain Cz safety in powdered milk and flour (Fig. 2). Observed negative correlation between HDI and sample size could suggest the need to improve on technical-know related to sample size and sampling training in Cz surveillance in milk and flour. Inverse correlation between SuDI and Cz positivity is indicative that improved, sustainable production and practices related to powdered milk and flour would generally enhance its safety. It suffices to say that the result aligned with SuDG 2, especially SuDG 2.1 that aimed to “end hunger and ensure access by all people, in particular the poor and people in vulnerable situations, including infants, to safe, nutritious and sufficient food all year round by 2030”, however, the inverse correlation between SuDI and sample size partly unveiled insufficient sampling plan in Cz monitoring and could partly undermined sustainable powdered milk and flour safety. On the otherwise, it suggests that sample size decreases with increasing SuDI.

The global prevalence of Cz in PMF was 8.39% coupled with a LOSOCV value of 7.66% was slightly higher than previously reported 8% (0.066–0.096) pooled global prevalence of *Cronobacter* species in animal originated sources including 1045 PIF, 96 follow formula, 182 powdered instant products, 175 milk powder, 92 pork, and 222 minced meat samples for the period 2008–2014 based on fixed effects model^110^(Sani and Odeyemi, 2015). Also, the present prevalence is higher than the pooled overall *Cronobacter* prevalence of 5% (0.001–0.038) in powdered instant products reported by Sani and Odeyemi^110^. Nonetheless, the previous report fell with the PI of Cz in powdered milk and flour in this study. The robustness of the current study hinged on the absence of small-study effects as presented by the Eggers' test and trim-fill results unlike the study of Sani and Odeyemi that reported presence of publication bias^110^. The observed high level of heterogeneity in this study is not surprising as there are subtle/obvious differences in the experimental design, samples, cultural setting (nation), detection methodology, spread and precision of prevalence estimates across the individual studies (Fig. 3, Table 3) and the absence of publication bias in this study probably indicated that research outputs on Cz prevalence in PMF get published irrespective of their favourable or unfavourable outcomes. Publication bias connotes failure to publish a study based on the weakness (statistically insignificant or negative studies) or strength (statistically significant weak results) of the study’s findings^111^.

The methodological approach in the determination of Cz contamination in powdered milk and flour varied significantly with highest prevalence achieved by PCR, followed by combination of C and PCR, C and API. It is well established that direct PCR is meritorious in assessing pathogens including culturable and viable but not culturable (VBNC) cells but, lack the ability to differentiate between living and dead cells^112,113^. Whereas the combination of culture and PCR solely accountable for culturable living cells and underestimate VBNC cells^112,113^. VBNC cells can establish infections and endangered food safety as well as the culturable cells^114,115^. Thus, underscore the need for methods that allowed holistic assessment of Cz in powdered milk and flours. Methods that rely on sequential application or combination of C and GN_VITEK2; C, GN_VITEK2 and PCR, C, and C, API, and PCR (Table 2) would invariably underestimate Cz prevalence in powdered milk and flour, owning to it capability to enter VBNC state because of desiccation stress under prolong storage. It is crucial that viable and VBNC Cz cells which may concomitantly exist in powdered milk samples be regarded in the design of survaillance activitis in term of sample preparation techniques**,** Cz identification method, and Cz culture techniques to shield against false negative results and insensitivity.

The type/variety of PMF did not significantly affect the prevalence of Cz in PMF with the highest recorded in EPIF (14.53%), followed by IFF (12.84%), CPIF (12.73%), Ifoods (8.92%), PIF (6.25%), and FUF (2.32%) (Table 2). Thus, the results emphasize diverse Cz exposure potential hubs via difference varieties of PMF.

The choice of appropriate procedural schemes in Cz monitoring in PMF is linked with the significant difference in Cz prevalence in powdered milk based on DNA extraction method in PMF. For instance, the use of kit in DNA extraction substantially resulted in a higher prevalence (10.69%) compared with boiling method (6.55%), this might be attributed to higher use of kits compared to boiling method in DNA extraction. It is unknown whether the sensitivity of boiling method of DNA extraction varied with pathogen species or not; thus, worthy of future research.

Creation and implementation of Cz monitoring program in PMF across regions irrespective of the socioeconomic statuses including HDI have become an urgent need as Cz prevalence in PMF was significantly difference across continents but not HDI (Table 2). Further neglects might result into the use of curative controls of Cz PMF-borne infections which are costly with high attending economic burden and unsustainable model unlike the preventive frameworks that could safegurd the public health including children, immunocompromised and immunocompetent individuals at very cheap cost. Clinical outcome of Cz infections can be variable, for instance, 2 pediatrics Cz cases in USA from PIF and maternal expressed milk led to one survival and one death^116^.

The Cz prevalence in PMF varied across WHO regions declining from 26.39% in AFR, 22.61% in AMR, 8.81% in WPR, 7.62% in EMR, 5.45% in EUR to 2.85% in SEAR (Table 2) in the same way it declined significantly from LATC (26.46%), EAP (8.81%), MENA (7.62%), ECA (5.45%), North America (4.46%) to South Asia (2.85%) among world bank regional classification. Thus indirectly revealed regional degree of action needed to monitor Cz. The lower the prevalence, sometimes indicate that survailance efforts are limited in the region, and not necessarily the occurrence of Cz in powdered milk/flaours in the various regions. This should guard intenvention funds and programme across the regions.

Individual studies from various regions reported Cz prevalence in PMF higher than the global prevalence. For instance, individual studies from Africa reported Cz prevalence in PMF as 0.00% in PIF^117^, 5.36%^118^, and 11.25%^10^ in Egypt; 0.00%^119^ (India, PIF), 1.10%^120^ (China, PIF), 1.45%^121^ (Jordan, PIF), 1.67%^11^ (China, PIF), 2.00%^7^ (China, PIF), 3.08%^9^ (Iraq, PIF), 3.13%^122^(Bangladesh, PIF), 4.07%^123^ (China, PIF), 4.12%^124^ (China, Ifoods), 4.34%^125^ (China, PIF), 5.00%^126^ (Iran, PIF), 6.04%^127^(Japan, PIF), 6.86%^127^(Korea, PIF), 7.20%^128^ (Iran, PIF), 7.51%^129^ (China, IFF), 8.00%^126^ (Iran, Ifoods), 10.60%^130^(China, EPIF), 11.94%^40^ (China, IFF), 12.28%^131^ (China, PIF), 16.53%^11^ (China, CPIF), 16.88%^132^ (China, PIF), and 47.67%^133^ (Korea, IFF) in Asia. In Europe, individuals studies demonstrated Cz prevalence in PMF as 0.00%^134^, Czech Republic, IFF/PIF; 0.00%^135^ (Turkey, CPIF), 0.00%^136^ (Ireland, PIF), 0.35%^107^ (Netherlands, PIF), 0.50%^137^ (Slovakia, PIF), 0.74%^138^ (UK, FUF), 1.00%^139^ (Germany, EPIF), 1.16%^137^(Slovakia, CPIF), 2.50%^136^ (Ireland, IFF), 3.33%^140^ (Czech Republic, PIF), 4.09%^141^ (Austria, PIF), 10.00%^142^ ( Turkey, PIF), 11.11%^140^ (Czech Republic, IFF), 12.29%^138^ (UK, Ifoods), 14.13%^139^ (Germany, PIF), 15.00%^142^ (Turkey, EPIF), 19.80%^143^ (Turkey, IFF), 39.71%^141^ (Austria, EPIF), 60.53%^144^ (France, EPIF), and 100.00%^145^ (Turkey, raw PIF); 4.46%^146^ (USA, EPIF) and 62.00%^147^ (Mexico, PIF) in North America, and 0.00%^148^ (Brazil, EPIF), 4.69%^149^ (Chile), 17.91%^148^ (Brazil, PIF), 23.33%^150^ (Brazil, CPIF), 34.31%^151^ (Colombia, IFF), 44.44%^152^ (Brazil, CPIF), and 66.67%^150^ (Brazil, IFF) from the South America. 

A number of factors singly or in combinations considerably influenced and predicted Cz prevalence in powdered milk and explained 0.48 to 70.30% (R^2^) of the Cz prevalence in PMF. In particular, nation, method, world bank region, WHO region, and N explained 53.88%, 19.62%, 19.03%, 15.63%, and 9.22% of the Cz prevalence in PMF, respectively. Thus, further corroborated the need for regional investment and methodological soundness for Cz monitoring of Cz in PMF. Also, bivariate interaction of N and Nation (R^2^ = 70.26%), milk type and Nation (R^2^ = 66.14%), N and Continent (R^2^ = 37.43%), milk type and world bank region (R^2^ = 34.67%), milk type and continent (R^2^ = 30.42%), N and WHO region (R^2^ = 29.54%), milk type and method (R^2^ = 28.53%), N and method (R^2^ = 26.14%), N and milk type (R^2^ = 22.61%), milk type and SuDI (R^2^ = 20.65%), and DNA extraction and SuDI (R^2^ = 19.11%) substantially accounted for the true differences in Cz prevalence in PMF. For nation and sample size to explain as high as 70.26% is an indication that national will power in the monitoring and surveillance of Cz in PMF with adequate sample size will go a long way in preventing Cz contamination, as well as the use of appropriate detection methods.

The limitations of the current study consisted in the inherent shortcomings of the included data. Also, the removal of studies/sub-studies with sample sizes < 10 and general scarcity of data from many countries prevented national based assessment of Cz prevalence in PMF which could have informed national priority and decisions. Thus, the listed inherent data limitations could impacts the outcomes and interpretations of the present study. However, the highlighted gaps could inform future research design.

## Conclusion

The present study revealed considerable association of sample-size with Cz positivity, Milkprod2020, and SuDI coupled with 8.39% (95%CI 6.06–11.51, PI: 0.46–64.35) global prevalence of Cz in PMF. Cz prevalence in PMF varies significantly with detection methods, DNA extraction method, across continents, WHO regions, and world bank regions. Nation, detection method, world bank region, WHO region, and sample size explained 53.88%, 19.62%, 19.03%, 15.63%, and 9.22% of the true differences in the Cz prevalence in PMF, respectively. However, Cz prevalence in PMF was negligibly difference across HDI and world bank income classes. Overall, the results indicated that national will power in the monitoring and surveillance of Cz in PMF matched with adequate sample size and appropriate detection methods will go a long way in preventing Cz contamination and subsequence infections.

### Supplementary Information

Supplementary Information.

## Data Availability

All data generated or analysed during this study are included in this published article and its supplementary information file.

## References

[1] Gurtler JB, Kornacki JL, Beuchat LR (2005). Enterobacter *sakazakii*: A coliform of increased concern to infant health. Int. J. Food Microbiol..

[2] Al-Nabulsi AA (2009). Influence of desiccation on the sensitivity of *Cronobacter* spp. to lactoferrin or nisin in broth and powdered infant formula. Int. J. Food Microbiol..

[3] Joseph S (2012). Comparative analysis of genome sequences covering the seven Cronobacter species. PLoS ONE.

[4] Lu Y (2019). Prevalence and genetic diversity of Cronobacter species isolated from four infant formula production factories in China. Front. Microbiol..

[5] Belal M, Al-Mariri A, Hallab L, Hamad I (2013). Detection of *Cronobacter* spp. (formerly *Enterobacter sakazakii*) from medicinal plants and spices in Syria. J. Infect. Dev. Ctries..

[6] Elkhawaga AA, Hetta HF, Osman NS, Hosni A, El-Mokhtar MA (2020). Emergence of *Cronobacter sakazakii* in cases of neonatal sepsis in upper Egypt: First report in North Africa. Front. Microbiol..

[7] Fei P (2018). Occurrence, genotyping, and antibiotic susceptibility of *Cronobacter* spp. in drinking water and food samples from Northeast China. J. Food Prot..

[8] Li C (2019). Prevalence, antibiotic susceptibility, and molecular characterization of *Cronobacter* spp. isolated from edible mushrooms in China. Front. Microbiol..

[9] Tayeb B, Mohamed Sharif Y, Ameen A (2020). Incidence rate and antibiotic resistance profile of *Cronobacter sakazakii* isolated from various food products. Food Res..

[10] Amer I, Mansour M, Abdelfatah E, Elshazely R (2020). *Cronobacter sakazakii* and microbiological profile of infant formulae and some dairy products consumed by infants. Adv. Anim. Vet. Sci.

[11] Li Y (2020). Prevalence and genetic characteristics of *Cronobacter* spp. from food and human clinical stool samples in Wenzhou, China 2008–2018. Food Microbiol..

[12] Yao K (2016). Isolation and characterization of *Cronobacter* spp. from indigenous infant flours sold in public health care centres within Abidjan, Côte d'Ivoire. Food Control.

[13] Zeng H (2020). Prevalence, genetic analysis and CRISPR typing of *Cronobacter* spp. isolated from meat and meat products in China. Int. J. Food Microbiol..

[14] Lou X (2019). The occurrence and distribution characteristics of *Cronobacter* in diverse cereal kernels, flour, and flour-based products. Food Microbiol..

[15] Lou X (2019). Potential reservoirs and routes of *Cronobacter* transmission during cereal growing, processing and consumption. Food Microbiol..

[16] Ogihara H (2019). Prevalence of *Cronobacter* spp. in retail foods and farm-associated environments in Japan. Food Sci. Technol. Res..

[17] Friedemann M (2007). *Enterobacter sakazakii* in food and beverages (other than infant formula and milk powder). Int. J. Food Microbiol..

[18] Venkitanarayanan MARAK (2011). Effect of trans-cinnamaldehyde on inhibition and inactivation of *Cronobacter sakazakii* biofilm on abiotic surfaces. J. Food Prot..

[19] Hunter C, Bean J (2013). Cronobacter: An emerging opportunistic pathogen associated with neonatal meningitis, sepsis and necrotizing enterocolitis. J. Perinatol..

[20] Ye Y (2014). Isolation and phenotypic characterization of *Cronobacter* from dried edible macrofungi samples. J. Food Sci..

[21] Iversen C, Forsythe S (2003). Risk profile of *Enterobacter sakazakii*, an emergent pathogen associated with infant milk formula. Trends Food Sci. Technol..

[22] Huang Y (2020). Inactivation efficacy of 405 nm LED against *Cronobacter sakazakii* biofilm. Front. Microbiol..

[23] Da Silva EP, De Martinis ECP (2013). Current knowledge and perspectives on biofilm formation: The case of Listeria monocytogenes. Appl. Microbiol. Biotechnol..

[24] Ha J-W, Kang D-H (2014). Synergistic bactericidal effect of simultaneous near-infrared radiant heating and UV radiation against *Cronobacter sakazakii* in powdered infant formula. Appl. Environ. Microbiol..

[25] Simões M, Simões LC, Vieira MJ (2010). A review of current and emergent biofilm control strategies. LWT-Food Sci. Technol..

[26] Harouna S (2015). Antibacterial activity of bovine milk lactoferrin on the emerging foodborne pathogen *Cronobacter sakazakii*: Effect of media and heat treatment. Food Control.

[27] Organization, W. H. *Enterobacter sakazakii and other microorganisms in powdered infant formula: meeting report*. (World health organization, 2004).

[28] Page MJ (2021). The PRISMA 2020 statement: An updated guideline for reporting systematic reviews. Int. J. Surg..

[29] Schwarzer G, Chemaitelly H, Abu-Raddad LJ, Rücker G (2019). Seriously misleading results using inverse of Freeman-Tukey double arcsine transformation in meta-analysis of single proportions. Res. Synth. Methods.

[30] Borenstein M, Higgins JP (2013). Meta-analysis and subgroups. Prev. Sci..

[31] Egger M, Smith GD, Schneider M, Minder C (1997). Bias in meta-analysis detected by a simple, graphical test. Bmj.

[32] Viechtbauer W (2010). Conducting meta-analyses in R with the metafor package. J. Stat. Softw..

[33] Viechtbauer, W., López-López, J. A., Sánchez-Meca, J. & Marín-Martínez, F. *A comparison of procedures to test for moderators in mixed-effects meta-regression models*. Vol. 20 (American Psychological Association, 2015). 10.1037/met0000023 25110905

[34] Good P (2013). Permutation Tests: A Practical Guide to Resampling Methods for Testing Hypotheses.

[35] Peterson, B. G. *et al.* Package ‘performanceanalytics’. *R Team Cooperation* **3**, 13–14 (2018).

[36] Harrer M (2020). Prevention of eating disorders at universities: A systematic review and meta-analysis. Int. J. Eat. Disord..

[37] Balduzzi S, Rücker G, Schwarzer G (2019). How to perform a meta-analysis with R: A practical tutorial. BMJ Ment. Health.

[38] Badawy B (2022). Prevalence and antimicrobial resistance of virulent listeria monocytogenes and *Cronobacter sakazakii* in dairy cattle, the environment, and dried milk with the in vitro application of natural alternative control. Antibiotics-Basel.

[39] Li Y (2020). Prevalence and genetic characteristics of *Cronobacter* spp. from food and human clinical stool samples in Wenzhou, China 2008–2018. Food Microbiol..

[40] Liang AL (2020). Molecular typing and drug resistance of *Cronobacter* spp. in commercial formula rice flour products for infants and young children. Mod. Food Sci. Technol..

[41] Ziver T, Okburan G, Akgül Ö, Sarıbaş S, Kocazeybek B (2020). Investigation of *Cronobacter sakazakii* (*Enterobacter sakazakii*) presence in cereal infant foods. Prog. Nutr..

[42] Costa PV (2020). Multi-locus sequence typing and antimicrobial susceptibility profile of *Cronobacter sakazakii* and *Cronobacter malonaticus* isolated from corn-based farinaceous foods commercialized in Brazil. Food Res. Int..

[43] Amer IH, Mansour MAH, Abdelfatah EN, Elshazely RMY (2020). *Cronobacter sakazakii* and microbiological profile of infant formulae and some dairy products consumed by infants. Adv. Anim. Vet. Sci..

[44] Tayeb BA, Mohamed Sharif YH, Ameen AM (2020). Incidence rate and antibiotic resistance profile of *Cronobacter sakazakii* isolated from various food products. Food Res..

[45] Hayman MM (2020). Prevalence of *Cronobacter* spp. and *Salmonella* in milk powder manufacturing facilities in the United States. J. Food Prot..

[46] Mashoufi A (2019). A novel primer targeted gyrB gene for the identification of *Cronobacter sakazakii* in powdered infant formulas (PIF) and baby foods in Iran. J. Food Saf..

[47] Peng FEI (2018). Occurrence, genotyping, and antibiotic susceptibility of *Cronobacter* spp. in drinking water and food samples from Northeast China. J. Food Prot..

[48] Demirci, Ü., Hakkı Tekiner, İ., Çakmak, B. & Özpınar, H. Occurrence and molecular characterization of different virulence-associated genes of *Cronobacter sakazakii* isolates from some foods and dust samples. *Ocorrência e caracterização molecular de diferentes genes associados à virulência de Cronobacter sakazakii detectados em alguns alimentos e amostras de poeira.* **48**, 1–9. 10.1590/0103-8478cr20180127 (2018).

[49] Tutar E, Akinci KS, Akyol I (2018). Development and application of a new multiplex real-time PCR assay for simultaneous identification of *Brucella melitensis*, *Cronobacter sakazakii* and Listeria monocytogenes in raw milk and cheese. Int. J. Dairy Technol..

[50] Morato-Rodriguez MD, Velandia-Rodriguez D, Castaneda S, Crosby M, Vera H (2018). *Cronobacter* spp. in common breast milk substitutes, Bogota, Colombia. Emerg. Infect. Dis..

[51] Zhang H (2017). Surveillance and molecular typing of *Cronobacter* spp. in commercial powdered infant formula and follow-up formula from 2011 to 2013 in Shandong Province, China. J. Sci. Food Agric..

[52] Brandão MLL, Umeda NS, Jackson E, Forsythe SJ, Filippis Id (2017). Isolation molecular and phenotypic characterization, and antibiotic susceptibility of *Cronobacter* spp. from Brazilian retail foods. Food Microbiol..

[53] Mardaneh J, Soltan Dallal MM (2016). Study of *Cronobacter sakazakii* strains isolated from powdered milk infant formula by phenotypic and molecular methods in Iran. Arch. Pediatr. Infect. Dis..

[54] Kakatkar AS, Gautam RK, Godambe LP, Shashidhar R (2017). Culture dependent and independent studies on emerging food-borne pathogens *Cronobacter sakazakii*, *Klebsiella pneumoniae* and *Enterococcus faecalis* in Indian food. Int. Food Res. J..

[55] Pei X (2016). The survey of *Cronobacter* spp. (formerly *Enterobacter sakazakii*) in infant and follow-up powdered formula in China in 2012. Biomed. Environ. Sci..

[56] Li Z (2016). Prevalence and characterization of *Cronobacter sakazakii* in retail milk-based infant and baby foods in Shaanxi, China. Foodborne Pathog. Dis..

[57] Aksu F, Sandikçi Altunatmaz S, Issa G, Özmen Togay S, Aksu H (2016). Prevalence and identification by multiplex polymerase chain reaction patterns of *Cronobacter* spp. isolated from plant-based foods. Food Sci. Technol. (Brazil).

[58] Parra-Flores, J., Rodriguez FernÁNdez, A., Contreras, FernÁNdez, A. & Aguirre GarcÍA, J. RIESGO DE ENFERMAR POR *Cronobacter sakazakii* ASOCIADO AL CONSUMO DE LECHES EN POLVO EN NIÑOS CHILENOS MENORES DE 2 AÑOS. *Risk of illness by *Cronobacter sakazakii* associated with powdered milk consumption in Chilean infants younger than 2 years of age.* **23**, S62–S63 (2016).

[59] Fang RY (2015). Prevalence and subtyping of *Cronobacter* species in goat milk powder factories in Shaanxi province, China. J. Dairy Sci..

[60] Huang Y (2015). Occurrence and characterization of *Cronobacter* spp. in dehydrated rice powder from Chinese supermarket. Plos ONE.

[61] Pan Z (2014). Isolation and molecular typing of *Cronobacter* spp. in commercial powdered infant formula and follow-up formula. Foodborne Pathog. Dis..

[62] Xu F (2014). Detection of *Cronobacter* species in powdered infant formula by probe-magnetic separation PCR. J. Dairy Sci..

[63] Mozrová V, Brenová N, Mrázek J, Lukesová D, Marounek M (2014). Surveillance and characterisation of *Cronobacter* spp. in Czech retail food and environmental samples. Folia Microbiol..

[64] Gicová A, Oriesková M, Oslanecová L, Drahovská H, Kaclíková E (2014). Identification and characterization of *Cronobacter* strains isolated from powdered infant foods. Lett. Appl. Microbiol..

[65] Siqueira Santos RF (2013). Screening for *Cronobacter* species in powdered and reconstituted infant formulas and from equipment used in formula preparation in maternity hospitals. Ann. Nutr. Metab..

[66] Hochel I, Růžičková H, Krásný L, Demnerová K (2012). Occurrence of *Cronobacter* spp. in retail foods. J. Appl. Microbiol..

[67] Jongenburger I, Reij MW, Boer EP, Gorris LG, Zwietering MH (2011). Actual distribution of *Cronobacter* spp. in industrial batches of powdered infant formula and consequences for performance of sampling strategies. Int. J. Food Microbiol..

[68] Oonaka K, Furuhata K, Hara M, Fukuyama M (2010). Powder Infant Formula Milk Contaminated with *Enterobacter sakazakii*. Jpn. J. Infect. Dis..

[69] Park JH, Lee YD, Ryu TW, Chang HI (2010). Identification and classification of *Cronobacter* spp. isolated from powdered food in Korea. J. Microbiol. Biotechnol..

[70] Reich F, König R, von Wiese W, Klein G (2010). Prevalence of *Cronobacter* spp. in a powdered infant formula processing environment. Int. J. Food Microbiol..

[71] Hoque A (2010). Isolation and molecular identification of Cronobacter spp from powdered infant formula (PIF) in Bangladesh. Int. J. Food Microbiol..

[72] Ye YW (2009). A comparison of polymerase chain reaction and international organization for standardization methods for determination of *Enterobacter sakazakii* contamination of infant formulas from chinese mainland markets. Foodborne Pathog. Dis..

[73] Chap J (2009). International survey of *Cronobacter sakazakii* and other *Cronobacter* spp. in follow up formulas and infant foods. Int. J. Food Microbiol..

[74] O'Brien S, Healy B, Negredo C, Fanning S, Iversen C (2009). Evaluation of a new one-step enrichment in conjunction with a chromogenic medium for the detection of *Cronobacter* spp. (*Enterobacter sakazakii*) in powdered infant formula. J. Food Prot..

[75] Hein I (2009). Temporal and spatial distribution of *Cronobacter* isolates in a milk powder processing plant determined by pulsed-field gel electrophoresis. Foodborne Pathog. Dis..

[76] El-Sharoud WM (2009). Characterization of *Cronobacter* recovered from dried milk and related products. BMC Microbiol..

[77] Jaradat ZW, Ababneh QO, Saadoun IM, Samara NA, Rashdan AM (2009). Isolation of *Cronobacter* spp. (formerly *Enterobacter sakazakii*) from infant food, herbs and environmental samples and the subsequent identification and confirmation of the isolates using biochemical, chromogenic assays, PCR and 16S rRNA sequencing. BMC Microbiol..

[78] Derzelle S (2007). Comparison of three chromogenic media and evaluation of two molecular-based identification systems for the detection of *Enterobacter sakazakii* irom environmental samples from infant formulae factories. J. Food Prot..

[79] Torres-Chavolla E, Ramírez-Cerda E, Gutiérrez-Rojo R (2007). Isolation and identification of *Enterobacter sakazakii* in infant milk formulas. Foodborne Pathog. Dis..

[80] Kaclíková E, Turcovský I (2011). A method for the detection of cronobacter strains in powdered milk-based foods using enrichment and real-time PCR. J. Food Nutr. Res..

[81] Kandhai MC, Reij MW, Gorris LGM, Guillaume-Gentil O, Van Schothorst M (2004). Occurrence of *Enterobacter sakazakii* in food production environments and households. Lancet.

[82] Gutiérrez-Rojo R, Torres-Chavolla E (2007). A rapid polymerase chain reaction assay for *Enterobacter sakazakii* detection in infant milk formulas. J. Rapid Methods Autom. Microbiol..

[83] Guillaume-Gentil O, Sonnard V, Kandhai MC, Marugg JD, Joosten H (2005). A simple and rapid cultural method for detection of *Enterobacter sakazakii* in environmental samples. J. Food Prot..

[84] Shaker R, Osaili T, Al-Omary W, Jaradat Z, Al-Zuby M (2007). Isolation of *Enterobacter sakazakii* and other *Enterobacter* sp. from food and food production environments. Food Control.

[85] Kandhai MC (2010). A study into the occurrence of *Cronobacter* spp. in The Netherlands between 2001 and 2005. Food Control.

[86] Lee YD, Park JH, Chang H (2012). Detection, antibiotic susceptibility and biofilm formation of *Cronobacter* spp. from various foods in Korea. Food Control.

[87] Zhou Y (2008). Development of an immobilization and detection method of *Enterobacter sakazakii* from powdered infant formula. Food Microbiol..

[88] Craven HM, McAuley CM, Duffy LL, Fegan N (2010). Distribution, prevalence and persistence of *Cronobacter* (*Enterobacter sakazakii*) in the nonprocessing and processing environments of five milk powder factories. J. Appl. Microbiol..

[89] Sani NA, Yi LY (2011). Enterobacteriaceae, *Cronobacter* (*Enterobacter*) *sakazakii* and microbial population in infant formula products in the Malaysian market. Sains Malays..

[90] Choi JW (2008). Multiple confirmation and RAPD-genotyping of *Enterobacter sakazakii* isolated from Sunsik. Korean J. Food Sci. Technol..

[91] Ragab NW, Abdelaziz SM, Galal SM, Abdelsabour E, Abdelhamid RF (2023). Identification of *Cronobacter sakazakii* isolated from powdered infant formula and stool of infants. Assiut Vet. Med. J. (Egypt).

[92] Lehner A, Fricker-Feer C, Gschwend K, Stephan R (2010). Identification of enterobacteriaceae and *Cronobacter* spp. in raw milk, milk concentrate and milk powder: Prevalence and genotyping. Archiv fur Lebensmittelhygiene.

[93] El-Gamal MS, El Dairouty RK, Okda AY, Salah SH, El-Shamy SM (2013). Incidence and interrelation of *Cronobacter sakazakii* and other foodborne bacteria in some milk products and infant formula milks in Cairo and Giza area. World Appl. Sci. J..

[94] Witthuhn RC, Kemp F, Britz TJ (2007). Isolation and PCR detection of *Enterobacter sakazakii* in South African food products, specifically infant formula milks. World J. Microbiol. Biotechnol..

[95] Iversen C, Forsythe S (2004). Isolation of *Enterobacter sakazakii* and other Enterobacteriaceae from powdered infant formula milk and related products. Food Microbiol..

[96] Aigbekaen BO, Oshoma CE (2010). Isolation of *Enterobacter sakazakii* from powdered foods locally consumed in Nigeria. Pak. J. Nutr..

[97] Li YH (2014). Isolation, identification and antimicrobial resistance of *Cronobacter* spp. isolated from various foods in China. Food Control.

[98] Choi SH, Choi JW, Lee SB (2008). Genotyping based on polymerase chain reaction of *Enterobacter sakazakii* isolates from powdered infant foods. Food Sci. Biotechnol..

[99] Lou X (2014). Possible reservoir and routes of transmission of *Cronobacter* (*Enterobacter sakazakii*) via wheat flour. Food Control.

[100] Gökmen M, Tekinşen KK, Gürbüz U (2010). Presence of *Enterobacter sakazakii* in milk powder, whey powder and white cheese produced in Konya. Kafkas Universitesi Veteriner Fakultesi Dergisi.

[101] Kim H, Bang J, Beuchat LR, Ryu JH (2008). Fate of *Enterobacter sakazakii* attached to or in biofilms on stainless steel upon exposure to various temperatures or relative humidities. J. Food Prot..

[102] Jung MK, Park JH (2006). Prevalence and thermal stability of *Enterobacter sakazakii* from unprocessed ready-to-eat agricultural products and powdered infant formulas. Food Sci. Biotechnol..

[103] Zhao YL (2010). Rapid and sensitive detection of *Enterobacter sakazakii* by cross-priming amplification combined with immuno-blotting analysis. Mol. Cell. Probes.

[104] Parra FJ (2015). Risk of *Cronobacter sakazakii* contamination in powdered milk for infant nutrition. Rev. Chilena de Nutricion.

[105] El-Sharoud WM, El-Din MZ, Ziada DM, Ahmed SF, Klena JD (2008). Surveillance and genotyping of *Enterobacter sakazakii* suggest its potential transmission from milk powder into imitation recombined soft cheese. J. Appl. Microbiol..

[106] Kim M, Reyes GA, Cheng X, Stasiewicz MJ (2023). Simulation evaluation of power of sampling plans to detect *Cronobacter* in powdered infant formula production. J. Food Protection.

[107] Jongenburger I, Reij M, Boer E, Gorris L, Zwietering M (2011). Actual distribution of *Cronobacter* spp. in industrial batches of powdered infant formula and consequences for performance of sampling strategies. Int. J. Food Microbiol..

[108] Valero A, Pasquali F, De Cesare A, Manfreda G (2014). Model approach to estimate the probability of accepting a lot of heterogeneously contaminated powdered food using different sampling strategies. Int. J. Food Microbiol..

[109] Kim M, Miller MJ, Stasiewicz MJ (2022). Perspective: Challenges with product testing in powdered infant formula. J. Dairy Sci..

[110] Sani NA, Odeyemi OA (2015). Occurrence and prevalence of *Cronobacter* spp. in plant and animal derived food sources: A systematic review and meta-analysis. Springerplus.

[111] Nair AS (2019). Publication bias: Importance of studies with negative results!. Indian J. Anaesth..

[112] Kohn B (1999). LISTERIA| Detection by Commercial Immunomagnetic Particle-Based Assays.

[113] Cudjoe KS (1999). Immunomagnetic Particle-Based Techniques: Overview.

[114] Yoon J-H, Lee S-Y (2022). Characteristics of viable-but-nonculturable *Vibrio parahaemolyticus* induced by nutrient-deficiency at cold temperature. Crit. Rev. Food Sci. Nutr..

[115] Liu J, Yang L, Kjellerup BV, Zhenbo X (2023). Viable but nonculturable (VBNC) state, an underestimated and controversial microbial survival strategy. Trends Microbiol..

[116] Haston JC (2023). *Cronobacter sakazakii* infections in two infants linked to powdered infant formula and breast pump equipment—United States, 2021 and 2022. Morb. Mortal. Wkly. Rep..

[117] Badawy B (2022). Prevalence and antimicrobial resistance of virulent Listeria monocytogenes and *Cronobacter sakazakii* in dairy cattle, the environment, and dried milk with the in vitro application of natural alternative control. Antibiotics.

[118] El-Sharoud WM (2009). Characterization of *Cronobacter* recovered from dried milk and related products. BMC Microbiol..

[119] Kakatkar A, Gautam R, Godambe PL, Shashidhar R (2017). Culture dependent and independent studies on emerging food-borne pathogens *Cronobacter sakazakii*, *Klebsiella pneumoniae* and *Enterococcus faecalis* in Indian food. Int. Food Res. J..

[120] Pei XY (2016). The survey of *Cronobacter* spp. (formerly *Enterbacter sakazakii*) in infant and follow-up powdered formula in China in 2012. Biomed. Environ. Sci..

[121] Jaradat ZW, Ababneh QO, Saadoun IM, Samara NA, Rashdan AM (2009). Isolation of *Cronobacter* spp. (formerly *Enterobacter sakazakii*) from infant food, herbs and environmental samples and the subsequent identification and confirmation of the isolates using biochemical, chromogenic assays, PCR and 16S rRNA sequencing. BMC Microbiol..

[122] Hoque A (2010). Isolation and molecular identification of *Cronobacter* spp. from powdered infant formula (PIF) in Bangladesh. Int. J. Food Microbiol..

[123] Zhang H (2017). Surveillance and molecular typing of *Cronobacter* spp. in commercial powdered infant formula and follow-up formula from 2011 to 2013 in Shandong Province, China. J. Sci. Food Agric..

[124] Ye Y (2009). A comparison of polymerase chain reaction and international organization for standardization methods for determination of *Enterobacter sakazakii* contamination of infant formulas from Chinese mainland markets. Foodborne Pathog. Dis..

[125] Xu X (2014). Occurrence and characterization of *Cronobacter* spp. in powdered formula from Chinese retail markets. Foodborne Pathog. Dis..

[126] Mashoufi A (2019). A novel primer targeted gyrB gene for the identification of *Cronobacter sakazakii* in powdered infant formulas (PIF) and baby foods in Iran. J. Food Saf..

[127] Oonaka K, Furuhata K, Hara M, Fukuyama M (2010). Powder infant formula milk contaminated with *Enterobacter sakazakii*. Jpn. J. Infect. Dis..

[128] Mardaneh J, Dallal MMS (2016). Study of *Cronobacter sakazakii* strains isolated from powdered milk infant formula by phenotypic and molecular methods in Iran. Arch. Pediatr. Infect. Dis..

[129] Huang Y (2015). Occurrence and characterization of *Cronobacter* spp. in dehydrated rice powder from Chinese supermarket. PLoS ONE.

[130] Fang R (2015). Prevalence and subtyping of *Cronobacter* species in goat milk powder factories in Shaanxi province, China. J. Dairy Sci..

[131] Pan Z (2014). Isolation and molecular typing of *Cronobacter* spp. in commercial powdered infant formula and follow-up formula. Foodborne Pathog. Dis..

[132] Li Z (2016). Prevalence and characterization of *Cronobacter sakazakii* in retail milk-based infant and baby foods in Shaanxi, China. Foodborne Pathog. Dis..

[133] Lee Y-D, Ryu T-W, Chang H-I, Park J-H (2010). Identification and classification of *Cronobacter* spp. isolated from powdered food in Korea. J. Microbiol. Biotechnol..

[134] Mozrová V, Břeňová N, Mrázek J, Lukešová D, Marounek M (2014). Surveillance and characterisation of *Cronobacter* spp. in Czech retail Food and environmental samples. Folia Microbiol..

[135] Ziver N, Okburan G, Akgül Ö, Saribas A, Kocazeybek B (2020). Investigation of *Cronobacter sakazakii* (*Enterobacter sakazakii*) presence in cereal infant foods. Prog. Nutr..

[136] O’Brien S (2009). Prevalence of *Cronobacter* species (*Enterobacter sakazakii*) in follow-on infant formulae and infant drinks. Lett. Appl. Microbiol..

[137] Gičová A, Oriešková M, Oslanecová L, Drahovská H, Kaclíková E (2014). Identification and characterization of *Cronobacter* strains isolated from powdered infant foods. Lett. Appl. Microbiol..

[138] Chap J (2009). International survey of *Cronobacter sakazakii* and other *Cronobacter* spp. in follow up formulas and infant foods. Int. J. Food Microbiol..

[139] Reich F, König R, Von Wiese W, Klein G (2010). Prevalence of *Cronobacter* spp. in a powdered infant formula processing environment. Int. J. Food Microbiol..

[140] Hochel I, Růžičková H, Krásný L, Demnerová K (2012). Occurrence of *Cronobacter* spp. in retail foods. J. Appl. Microbiol..

[141] Hein I (2009). Temporal and spatial distribution of *Cronobacter* isolates in a milk powder processing plant determined by pulsed-field gel electrophoresis. Foodborne Pathog. Dis..

[142] Demirci Ü, Tekiner İH, Çakmak B, Özpınar H (2018). Occurrence and molecular characterization of different virulence-associated genes of *Cronobacter sakazakii* isolates from some foods and dust samples. Ciência Rural.

[143] Aksu F, Sandikçi Altunatmaz S, Issa G, Özmen Togay S, Aksu H (2016). Prevalence and identification by multiplex polymerase chain reaction patterns of *Cronobacter* spp. isolated from plant-based foods. Food Sci. Technol..

[144] Derzelle S (2007). Comparison of three chromogenic media and evaluation of two molecular-based identification systems for the detection of Enterobacter sakazakii from environmental samples from infant formulae factories. J. Food Prot..

[145] Tutar E, Akıncı KS, Akyol İ (2018). Development and application of a new multiplex real-time PCR assay for simultaneous identification of *Brucella melitensis*, *Cronobacter sakazakii* and Listeria monocytogenes in raw milk and cheese. Int. J. Dairy Technol..

[146] Hayman MM (2020). Prevalence of *Cronobacter* spp. and *Salmonella* in milk powder manufacturing facilities in the United States. J. Food Prot..

[147] Torres-Chavolla E, Ramírez-Cerda E, Gutiérrez-Rojo R (2007). Isolation and identification of Enterobacter sakazakii in infant milk formulas. Foodborne Pathog. Dis..

[148] Siqueira Santos RF (2013). Screening for *Cronobacter* species in powdered and reconstituted infant formulas and from equipment used in formula preparation in maternity hospitals. Ann. Nutr. Metab..

[149] Parra-Flores J (2015). Investigation on the factors affecting *Cronobacter sakazakii* contamination levels in reconstituted powdered infant formula. Front. Pediatr..

[150] Brandão MLL, Umeda NS, Jackson E, Forsythe SJ, de Filippis I (2017). Isolation, molecular and phenotypic characterization, and antibiotic susceptibility of *Cronobacter* spp. from Brazilian retail foods. Food Microbiol..

[151] del Rocío Morato-Rodríguez M, Velandia-Rodríguez D, Castañeda S, Crosby M, Vera H (2018). Cronobacter spp. in common breast milk substitutes, Bogotá, Colombia. Emerg. Infect. Dis..

[152] Costa PV (2020). Multi-locus sequence typing and antimicrobial susceptibility profile of *Cronobacter sakazakii* and *Cronobacter malonaticus* isolated from corn-based farinaceous foods commercialized in Brazil. Food Res. Int..

