# Supplementary material for: Global and regional prevalence of Cronobacter sakazakii in powdered milk and flour
Source: Sci Rep. 2024 Mar 22;14:6865. doi: 10.1038/s41598-024-57586-x (PMC10957878; doi:10.1038/s41598-024-57586-x)
Supplement: Supplementary file 1 — Supplementary Information. [file 41598_2024_57586_MOESM1_ESM.docx]

**Supplementary materials for “Global and regional prevalence of *Cronobacter sakazakii* in powdered milk/flour: Insights from** **random-intercept logistic and mixed-effects regressions”**

**Supplementary methodology**

**Search terms and results**

1. PubMed
(cronobacter*[ti] | sakazakii*[ti]) & (flour*[tiab] | powder*[tiab] | formula*[tiab]) NOT review [ptyp]
**[478](tel:478) results**

**PubMed update** Tuesday, January 02, 2024 2:48:31 PM

| Search: **(cronobacter*[ti] \| sakazakii*[ti]) & (flour*[tiab] \| powder*[tiab] \| formula*[tiab]) AND 2023/03/07:2023/12/31[pdat] NOT review [ptyp]**  (("cronobacter*"[Title] OR "sakazakii*"[Title]) AND ("flour*"[Title/Abstract] OR "powder*"[Title/Abstract] OR "formula*"[Title/Abstract]) AND 2023/03/07:2023/12/31[Date - Publication]) NOT "review"[Publication Type] | [25](https://pubmed.ncbi.nlm.nih.gov/?term=%28cronobacter%2A%5Bti%5D+%7C+sakazakii%2A%5Bti%5D%29+%26+%28flour%2A%5Btiab%5D+%7C+powder%2A%5Btiab%5D+%7C+formula%2A%5Btiab%5D%29+AND+2023%2F03%2F07%3A2023%2F12%2F31%5Bpdat%5D+NOT+review+%5Bptyp%5D&size=200&sort=relevance) results |
| --- | --- |

**2. Scopus**

[**271**](tel:271)**document results**
( TITLE ( ( cronobacter*  OR  sakazakii ) )  AND  TITLE-ABS-KEY ( ( flour*  OR  powder*  OR  formula* ) ) )  AND  PUBYEAR  >  [2015](tel:2015)  AND  ( LIMIT-TO ( DOCTYPE ,  "ar" ) )

**Scopus update:**

( ( ( TITLE ( ( cronobacter* OR sakazakii ) ) AND TITLE-ABS-KEY ( ( flour* OR powder* OR formula* ) ) ) AND PUBYEAR < 2016 ) OR ( ( TITLE ( ( cronobacter* OR sakazakii ) ) AND TITLE-ABS-KEY ( ( flour* OR powder* OR formula* ) ) ) AND PUBYEAR = 2023 ) ) AND ( LIMIT-TO ( DOCTYPE , "ar" ) )

419 documents found

**3. WOS**

Search: Cronobacter* OR sakazakii (Title) AND (flour* OR powder* OR milk* OR formula*) (Topic) AND 2016-01-01/2023-03-07 (Publication Date) and Article (Document Types) Date

Run: Tue Mar 07 2023 11:00:21 GMT+0100 (West Africa Standard Time)

**Results:** **318**

**WOS update**

TI=(Cronobacter* OR sakazakii) AND TS=((flour* OR powder* OR milk* OR formula*)) AND (DOP=(1985-01-01/2015-12-31) OR DOP=(2023-03-08/2023-12-31)) AND DT=(Article)

Date Run: Tue Jan 02 2024 17:06:17 GMT+0200 (South Africa Standard Time)

**Results: 479**

**4. EBSCOhost**
Search Results: [451 - 469](tel:451%20-%20469) of [469](tel:469)

| Tuesday, March 07, 2023 11:40:09 AM |
| --- |

| **#** | **Query** | **Limiters/Expanders** | **Last Run Via** | **Results** |
| --- | --- | --- | --- | --- |
| S1 | TI(Cronobacter* OR sakazakii*) AND ((TI (Flour* OR powder* OR formula*)) OR (AB (Flour* OR powder* OR formula*))) | Limiters - Peer Reviewed; Publication Type: Academic Journal; Document Type: Article; Publication Type: Journal Article; Publication Type: Peer Reviewed Journal; Document Type: Journal Article; Publication Type: Article; Hidden NetLibrary Holdings; Publication Type: Academic Journal; Document Type: Article; Publication Type: Academic Journal; Research Article; Publication Type: Journal Article; Publication Type: Academic Journal; Document Type: Article; Journal or Document: Journal Article (EJ); Publication Type: Journal Articles; Document Type: Article; Publication Type: Academic Journal; Publication Type: Academic Journal; Document Type: Article; Publication Type: Academic Journal; Document Type: Article; Publication Type: Academic Journal; Document Type: Article; Publication Type: Primary Source Document; Document Type: Article; Publication Type: Journal Article; Document Type: Article; Document Type: Article; Document Type: Article; Publication Type: Academic Journal; Document Type: Article Expanders - Apply equivalent subjects Search modes - Boolean/Phrase | Interface - EBSCOhost Research Databases Search Screen - Advanced Search Database - Academic Search Ultimate;Agricola;APA PsycInfo;AtlaSerials, Religion Collection;Audiobook Collection (EBSCOhost);Business Source Ultimate;CAB Abstracts​​​​​​​ with Full Text;CINAHL with Full Text;Communication & Mass Media Complete;eBook Collection (EBSCOhost);ERIC;Fish, Fisheries & Aquatic Biodiversity Worldwide;Global Health;GreenFILE;Health Source - Consumer Edition;Health Source: Nursing/Academic Edition;Library, Information Science & Technology Abstracts;MasterFILE Premier;MasterFILE Reference eBook Collection;MEDLINE with Full Text;Newspaper Source;Regional Business News;SocINDEX with Full Text;SPORTDiscus with Full Text;Teacher Reference Center | 1,745 |

**EBSCOhost update** on Tuesday, January 02, 2024 2:48:31 PM

# Print Search History

|  | Tuesday, January 02, 2024 2:48:31 PM |
| --- | --- |

| **Query** | **Limiters/Expanders** | **Last Run Via** | **Results** |
| --- | --- | --- | --- |
| TI(Cronobacter* OR sakazakii*) AND ((TI (Flour* OR powder* OR formula*)) OR (AB (Flour* OR powder* OR formula*))) | Limiters - Peer Reviewed; Publication Date: 20230101-20231231; Publication Type: Academic Journal; Document Type: Journal article; Publication Type: Journal Article; Publication Type: Peer Reviewed Journal; Document Type: Journal Article; Publication Type: Article; Hidden NetLibrary Holdings; Publication Type: Academic Journal; Document Type: Article; Publication Type: Academic Journal; Research Article; Publication Type: Journal Article; Publication Type: Academic Journal; Document Type: Article; Journal or Document: Journal Article (EJ); Publication Type: Journal Articles; Publication Type: Journal Article; Document Type: Article; Publication Type: Academic Journal; Publication Type: Academic Journal; Document Type: Article; Publication Type: Academic Journal; Document Type: Article; Publication Type: Academic Journal; Document Type: Article; Publication Type: Primary Source Document; Document Type: Article; Document Type: Article; Document Type: Article; Document Type: Article; Publication Type: Academic Journal; Document Type: Article Expanders - Apply equivalent subjects Search modes - Boolean/Phrase | Interface - EBSCOhost Research Databases Search Screen - Advanced Search Database - Academic Search Ultimate;Agricola;APA PsycInfo;AtlaSerials, Religion Collection;Audiobook Collection (EBSCOhost);Business Source Ultimate;CAB Abstracts​​​​​​​ with Full Text;CINAHL with Full Text;Communication & Mass Media Complete;eBook Collection (EBSCOhost);ERIC;Fish, Fisheries & Aquatic Biodiversity Worldwide;Global Health;GreenFILE;Health Source - Consumer Edition;Health Source: Nursing/Academic Edition;Library, Information Science & Technology Abstracts;MasterFILE Premier;MasterFILE Reference eBook Collection;Newspaper Source;Regional Business News;SocINDEX with Full Text;SPORTDiscus with Full Text;Teacher Reference Center | 26 |

Infant formula flour (IFF)/ Flour: flour/instant dry soup samples/wheat-based infant food, potato dumpling powder; dehydrated rice powder (DRP), infant rice powder (IRF); breast milk substitutes(corn starch, plantain starch, other starches); IMF//powdered porridges/custards; soy-based infant formulae

PIF: PIF/milk-based: prefinal product/prepackaged final product/final packaged product/; infant formula milk powder (IFMP); Dried Milk/; FUF// Dried milk (Full-fat milk powder, Skimmed milk powder, Dried whey, Dried ice-cream, Dried artificial cream Sahlab, Infant milk formulas; milk powder;

FUF , powder adult formula (PAF//CPIF:

/Ifoods

CPIF: cereal-based powdered weaning food products/**cereal based infant formulas and complementary foods; cereal mixes for children;** corn-based farinaceous food

EPIF: environ. Samples from PIF factories; infant formulae factories, dust; goat powder milk facilities, Env. Sample Milk Powder Manufacturing Facilities, utensils (feeding bottles , bottles containing thickened cow’s milk, used feeding bottles, bottle brushes, dosing cups, bottle storage equipment and blenders), environ.samples

Where PCR used only for speciation, method was assigned to C. all different PCR such RTPCR, qPCR was assigned PCR. For Hochel et al. 2012 (Czech Republic) who utilized Culture, ENTERO24Kit, API32E, and MALDI-TOF MS, we assigned MALDI-TOF MS since is the only gold standard used in determine Cronobacter occurrence in the sample. All forms of commercial biochemical test (API20E, API32E, and ENTERO24 were assigned/grouped as API. Aigbekaen and Oshoma, 2010, 20/70 powdered infant foods, 13/50 powdered milk and 5/20 milk based products assigned to PIF. Zhao et al. 2010 (China, 236 milk powder products, Kandhai et al. 2010 (Netherlands, milk powders (7/175)

**Supplementary results**

**Identification of studies via databases**

Records removed *before screening*:

Duplicate records removed

(n =3187 )

Records identified from*:

Databases (**N =** **3,761**):

- PubMed = 503
- WOS = 797
- Scopus = 690
- EBSCOhost=1771

**Identification**

Records screened

(n = 574)

Records excluded

(n =**494** )

Reports sought for retrieval

(n =**80** )

Reports not retrieved

(n = 0)

**Screening**

Reports assessed for eligibility

(n =80 )

Reports excluded:

n=12*

Studies included in systematic review (n=68) and disaggregated into 88 sub-studies in the models

**Included**

**Figure S1. Schema for selecting studies on *Cz* in PMF.**

*Not powdered dried foods (n=2, Ogihara et al 2014; Chon et al 2012), artificially contaminated (n=1; Reij et al 2009), sample not collected/MLSA study (n =1; Kuhnert et al 2009); Cronobacter sp (n=1; Ruan et al 2013), and Full text unavailable (n= 6; Guner et al 2011; Magalhaes et al 2012; Wang et al 2015; Raghav and Aggarwal 2007; Fu et al 2011), and (Li et al 2023 (ex12/67), Sani et al 2014 available methodologic description only pointed out to Cronobacter species. It is difficult to ascertain that Cz was evaluated. Because of the high risk of reporting bias, the article was unanimously agreed to be excluded from analysis.

**Table** **Quality assessement tool for nonhuman-based or food-based prevalence studies.**

| **To assess risk of bias related to the purpose, data collection, methodological, and statistical soundness of nonhuman-based or food-based prevalence studies** | | | |
| --- | --- | --- | --- |
| Major Components | Response options | | |
| 1. Does the title speaks directly to the present subject being addressed? | No (0 point) | Somehow (1 point) | Yes (2 point) |
| 2. Is the aim clearly stated and relevant to the present study | Unclear and irrelevant (0 point) | Clearly stated but somehow relevant (1 point) | Clearly stated and relevant (2 point) |
| 3. Do results reported in abstract matched those ones within the text? | Unmatched (0 point) |  | Matched (2 point) |
| 4. Is the description of sampling plan adequate, accompany with date and sample handling information? | No (0 point) | Somehow but inadequate (lack sampling period /sample handling information) (1 point) | Adequately reported with sampling period and sample handling information (2 point) |
| 5. Is the sample size stated or computable from reported data in the study? | No (0 point) | Somehow but inadequate (1 point) | Yes (2 point) |
| 6. Is the sample size adequate (≥50arbitrary selected)? | <50 (0 point) | ≥50 or <100 (1 point) | ≥100 (2 point) |
| 7. Is the endpoints appropriate to achieve the aim of the study? | No (0 point) | Somehow but inadequate (1 point) | Yes and adequate (2 point) |
| 8. Does the method assessed the study endpoint (outcome) qualitatively or quantitatively in an unbiased manner? | Yes, but with high possibility of misidentification (1 point) | Somehow but slight possibility of misidentification (2 point)  C_API/ C_GN_VITEK2 | Yes, and adequate (3 point)  C_GN_VITEK2_PCR, PCR  C_API_PCR, C_PCR, C_PCR_CPA |
| 9.Is the choice of statistical analysis adequate? Mainly descriptive | Not reported (0 point) | Somehow but inadequate (1 point) | Yes and adequate (2 point) |

Total score >18 = low; 14 – 18 = moderate; <14 = high

Table: Quality of the data included in Cz prevalence in PMF.

| SN | Author | Item 1 | Item 2 | Item 3 | Item 4 | Item 5 | Item 6 | Item 7 | Item 8 | Item 9 | Total score | Bias level |
| --- | --- | --- | --- | --- | --- | --- | --- | --- | --- | --- | --- | --- |
| 1 | Badawy et al. 2022 (Egypt, PIF) | 2 | 2 | 2 | 2 | 2 | 2 | 2 | 3 | 2 | 19 | low risk |
| 2 | Y. Li et al. 2020 (China, PIF) | 2 | 2 | 2 | 2 | 2 | 2 | 2 | 1 | 2 | 17 | moderate risk |
| 3 | Y. Li et al. 2020 (China, CPIF) | 2 | 2 | 2 | 2 | 2 | 2 | 2 | 1 | 2 | 17 | moderate risk |
| 4 | Liang et al. 2020 (China, IFF) | 2 | 2 | 2 | 2 | 2 | 2 | 2 | 3 | 2 | 19 | low risk |
| 5 | Ziver et al. 2020 (Turkey, CPIF) | 2 | 2 | 2 | 2 | 2 | 2 | 2 | 1 | 2 | 17 | moderate risk |
| 6 | Costa et al. 2020 (Brazil, CPIF) | 2 | 2 | 2 | 2 | 2 | 0 | 2 | 3 | 2 | 17 | moderate risk |
| 7 | Amer et al. 2020 (Egypt, PIF) | 2 | 2 | 2 | 2 | 2 | 1 | 2 | 1 | 2 | 16 | moderate risk |
| 8 | Tayeb et al. 2020 (Iraq, PIF) | 2 | 2 | 2 | 2 | 2 | 2 | 2 | 3 | 2 | 19 | low risk |
| 9 | Hayman et al. 2020 (USA, EPIF) | 2 | 2 | 2 | 2 | 2 | 2 | 2 | 2 | 2 | 18 | moderate risk |
| 10 | Mashoufi et al. 2019 (Iran, PIF) | 2 | 2 | 2 | 2 | 2 | 2 | 2 | 3 | 2 | 19 | low risk |
| 11 | Mashoufi et al. 2019 (Iran, Ifoods) | 2 | 2 | 2 | 2 | 2 | 2 | 2 | 3 | 2 | 19 | low risk |
| 12 | Peng et al. 2018 (China, PIF) | 2 | 2 | 2 | 2 | 2 | 2 | 2 | 2 | 2 | 18 | moderate risk |
| 13 | Demirci et al. 2018 (Turkey, PIF) | 2 | 2 | 2 | 2 | 2 | 2 | 2 | 2 | 2 | 18 | moderate risk |
| 14 | Demirci et al. 2018 (Turkey, EPIF) | 2 | 2 | 2 | 2 | 2 | 0 | 2 | 2 | 2 | 16 | moderate risk |
| 15 | Tutar et al. 2018 (Turkey, PIF) | 2 | 2 | 2 | 2 | 2 | 0 | 2 | 3 | 2 | 17 | moderate risk |
| 16 | Morato-Rodriguez et al. 2018 (Colombia, IFF) | 2 | 2 | 2 | 2 | 2 | 2 | 2 | 3 | 2 | 19 | low risk |
| 17 | Zhang et al. 2017 (China, PIF) | 2 | 2 | 2 | 2 | 2 | 2 | 2 | 2 | 2 | 18 | moderate risk |
| 18 | Brandao et al. 2017 (Brazil, IFF) | 2 | 2 | 2 | 2 | 2 | 0 | 2 | 3 | 2 | 17 | moderate risk |
| 19 | Brandao et al. 2017 (Brazil, CPIF) | 2 | 2 | 2 | 2 | 2 | 0 | 2 | 3 | 2 | 17 | moderate risk |
| 20 | Mardaneh and Soltan 2017 (Iran, PIF) | 2 | 2 | 2 | 2 | 2 | 2 | 2 | 3 | 2 | 19 | low risk |
| 21 | Kakatkar et al. 2017 (India, PIF) | 2 | 2 | 2 | 2 | 2 | 0 | 2 | 3 | 2 | 17 | moderate risk |
| 22 | Pei et al. 2016 (China, PIF) | 2 | 2 | 2 | 2 | 2 | 2 | 2 | 1 | 2 | 17 | moderate risk |
| 23 | Z. Li et al. 2016 (China, PIF) | 2 | 2 | 2 | 2 | 2 | 2 | 2 | 2 | 2 | 18 | moderate risk |
| 24 | Aksu et al. 2016 (Turkey, IFF) | 2 | 2 | 2 | 2 | 2 | 2 | 2 | 3 | 2 | 19 | low risk |
| 25 | Parra-Flores et al. 2016 (Chile, PIF) | 2 | 2 | 2 | 2 | 2 | 2 | 2 | 2 | 2 | 18 | moderate risk |
| 26 | Fang et al. 2015 (China, EPIF) | 2 | 2 | 2 | 2 | 2 | 2 | 2 | 2 | 2 | 18 | moderate risk |
| 27 | Huang et al. 2015 (China, IFF) | 2 | 2 | 2 | 2 | 2 | 2 | 2 | 3 | 2 | 19 | low risk |
| 28 | Pan et al. 2014 (China, PIF) | 2 | 2 | 2 | 2 | 2 | 2 | 2 | 2 | 2 | 18 | moderate risk |
| 29 | Xu et al. 2014 (China, PIF) | 2 | 2 | 2 | 2 | 2 | 2 | 2 | 3 | 2 | 19 | low risk |
| 30 | Mozrova et al. 2014 (Czech Republic, PIF) | 2 | 2 | 2 | 2 | 2 | 0 | 2 | 3 | 2 | 17 | moderate risk |
| 31 | Mozrova et al. 2014 (Czech Republic, IFF) | 2 | 2 | 2 | 2 | 2 | 0 | 2 | 3 | 2 | 17 | moderate risk |
| 32 | Gicova et al. 2014 (Slovakia, PIF) | 2 | 2 | 2 | 2 | 2 | 2 | 2 | 3 | 2 | 19 | low risk |
| 33 | Gicova et al. 2014 (Slovakia, CPIF) | 2 | 2 | 2 | 2 | 2 | 2 | 2 | 3 | 2 | 19 | low risk |
| 34 | Siqueira-Santos et al. 2013 (Brazil, PIF) | 2 | 2 | 2 | 2 | 2 | 1 | 2 | 1 | 2 | 16 | moderate risk |
| 35 | Siqueira-Santos et al. 2013 (Brazil, EPIF) | 2 | 2 | 2 | 2 | 2 | 0 | 2 | 1 | 2 | 15 | moderate risk |
| 36 | Hochel et al. 2012 (Czech Republic, PIF) | 2 | 2 | 2 | 2 | 2 | 1 | 2 | 2 | 2 | 17 | moderate risk |
| 37 | Hochel et al. 2012 (Czech Republic, IFF) | 2 | 2 | 2 | 2 | 2 | 1 | 2 | 2 | 2 | 17 | moderate risk |
| 38 | Jongenburger et al. 2011 (Netherlands, PIF) | 2 | 2 | 2 | 2 | 2 | 2 | 2 | 1 | 2 | 17 | moderate risk |
| 39 | Oonaka et al. 2010 (Japan, PIF) | 2 | 2 | 2 | 2 | 2 | 2 | 2 | 1 | 2 | 17 | moderate risk |
| 40 | Park et al. 2010 (Korea, PIF) | 2 | 2 | 2 | 2 | 2 | 2 | 2 | 3 | 2 | 19 | low risk |
| 41 | Park et al. 2010 (Korea, IFF) | 2 | 2 | 2 | 2 | 2 | 1 | 2 | 3 | 2 | 18 | moderate risk |
| 42 | Reich et al. 2010 (Germany, PIF) | 2 | 2 | 2 | 2 | 2 | 2 | 2 | 1 | 2 | 17 | moderate risk |
| 43 | Reich et al. 2010 (Germany, EPIF) | 2 | 2 | 2 | 2 | 2 | 2 | 2 | 1 | 2 | 17 | moderate risk |
| 44 | Hoque et al. 2010 (Bangladesh, PIF) | 2 | 2 | 2 | 2 | 2 | 0 | 2 | 3 | 2 | 17 | moderate risk |
| 45 | Ye et al. 2009 (China, Ifoods) | 2 | 2 | 2 | 2 | 2 | 2 | 2 | 3 | 2 | 19 | low risk |
| 46 | Chap et al. 2009 (UK, FUF) | 2 | 2 | 2 | 2 | 2 | 2 | 2 | 1 | 2 | 17 | moderate risk |
| 47 | Chap et al. 2009 (UK, Ifoods) | 2 | 2 | 2 | 2 | 2 | 2 | 2 | 1 | 2 | 17 | moderate risk |
| 48 | OBrien et al. 2009 (Ireland, PIF) | 2 | 2 | 2 | 2 | 2 | 2 | 2 | 2 | 2 | 18 | moderate risk |
| 49 | OBrien et al. 2009 (Ireland, IFF) | 2 | 2 | 2 | 2 | 2 | 1 | 2 | 2 | 2 | 17 | moderate risk |
| 50 | Hein et al. 2009 (Austria, PIF) | 2 | 2 | 2 | 2 | 2 | 2 | 2 | 3 | 2 | 19 | low risk |
| 51 | Hein et al. 2009 (Austria, EPIF) | 2 | 2 | 2 | 2 | 2 | 2 | 2 | 3 | 2 | 19 | low risk |
| 52 | El-Sharoud et al. 2009 (Egypt, PIF) | 2 | 2 | 2 | 2 | 2 | 2 | 2 | 3 | 2 | 19 | low risk |
| 53 | Jaradat et al. 2009 (Jordan, PIF) | 2 | 2 | 2 | 2 | 2 | 1 | 2 | 3 | 2 | 18 | moderate risk |
| 54 | Derzelle et al. 2007 (France, EPIF) | 2 | 2 | 2 | 2 | 2 | 0 | 2 | 3 | 2 | 17 | moderate risk |
| 55 | Torres-Chavolla et al. 2007 (Mexico, PIF) | 2 | 2 | 2 | 2 | 2 | 1 | 2 | 2 | 2 | 17 | moderate risk |
| 56 | Kaclikova and Turcovsky 2011(Slovakia, PIF) | 2 | 2 | 2 | 2 | 2 | 0 | 2 | 3 | 2 | 17 | moderate risk |
| 57 | Kaclikova and Turcovsky 2011(Slovakia, Ifoods) | 2 | 2 | 2 | 2 | 2 | 0 | 2 | 3 | 2 | 17 | moderate risk |
| 58 | Kandhai et al. 2004 (Netherlands, EPIF) | 2 | 2 | 2 | 2 | 2 | 2 | 2 | 2 | 2 | 18 | moderate risk |
| 59 | Gutierrez-Rojo and Torres-Chavolla 2007 (Mexico, PIF) | 2 | 2 | 2 | 2 | 2 | 1 | 2 | 3 | 2 | 18 | moderate risk |
| 60 | Guillaume-Gentil et al. 2005 (Netherlands, EPIF) | 2 | 2 | 2 | 2 | 2 | 2 | 2 | 2 | 2 | 18 | moderate risk |
| 61 | Shaker et al. 2007 (Jordan, CPIF) | 2 | 2 | 2 | 2 | 2 | 0 | 2 | 2 | 2 | 16 | moderate risk |
| 62 | Shaker et al. 2007 (Jordan, IFF) | 2 | 2 | 2 | 2 | 2 | 0 | 2 | 2 | 2 | 16 | moderate risk |
| 63 | Kandhai et al. 2010 (Netherlands, PIF) | 2 | 2 | 2 | 2 | 2 | 2 | 2 | 3 | 2 | 19 | low risk |
| 64 | Kandhai et al. 2010 (Netherlands, IFF) | 2 | 2 | 2 | 2 | 2 | 2 | 2 | 3 | 2 | 19 | low risk |
| 65 | Lee et al. 2012 (South Korea, Ifoods) | 2 | 2 | 2 | 2 | 2 | 1 | 2 | 3 | 2 | 18 | moderate risk |
| 66 | Zhou et al. 2008 (China, PIF) | 2 | 2 | 2 | 2 | 2 | 0 | 2 | 3 | 2 | 17 | moderate risk |
| 67 | Craven et al. 2010 (Australia, EPIF) | 2 | 2 | 2 | 2 | 2 | 2 | 2 | 2 | 2 | 18 | moderate risk |
| 68 | Sani and Yi 2011(Malaysia, PIF) | 2 | 2 | 2 | 2 | 2 | 1 | 2 | 2 | 2 | 17 | moderate risk |
| 69 | Choi et al. 2008 (South Korea, CPIF) | 2 | 2 | 2 | 2 | 2 | 1 | 2 | 2 | 2 | 17 | moderate risk |
| 70 | Choi et al. 2008 (South Korea, PIF) | 2 | 2 | 2 | 2 | 2 | 0 | 2 | 2 | 2 | 16 | moderate risk |
| 71 | Ragab et al. 2023 (Egypt, PIF) | 2 | 2 | 2 | 2 | 2 | 1 | 2 | 3 | 2 | 18 | moderate risk |
| 72 | Lehner et al. 2010 (Switzerland, PIF) | 2 | 2 | 2 | 2 | 2 | 2 | 2 | 3 | 2 | 19 | low risk |
| 73 | El-Gamal et al. 2013 (Egypt, PIF) | 2 | 2 | 2 | 2 | 2 | 1 | 2 | 1 | 2 | 16 | moderate risk |
| 74 | Witthuhn et al. 2007 (PIF, South Africa) | 2 | 2 | 2 | 2 | 2 | 0 | 2 | 3 | 2 | 17 | moderate risk |
| 75 | Iversen and Forsythe 2004 (UK, PIF) | 2 | 2 | 2 | 2 | 2 | 2 | 2 | 1 | 2 | 17 | moderate risk |
| 76 | Aigbekaen and Oshoma, 2010 (Nigeria, PIF) | 2 | 2 | 2 | 2 | 2 | 1 | 2 | 1 | 2 | 16 | moderate risk |
| 77 | Li et al. 2014 (China, CPIF) | 2 | 2 | 2 | 2 | 2 | 1 | 2 | 1 | 2 | 16 | moderate risk |
| 78 | Li et al. 2014 (China, PIF) | 2 | 2 | 2 | 2 | 2 | 0 | 2 | 1 | 2 | 15 | moderate risk |
| 79 | Li et al. 2014 (China, IFF) | 2 | 2 | 2 | 2 | 2 | 0 | 2 | 1 | 2 | 15 | moderate risk |
| 80 | Choi et al. 2008b (South Korea, CPIF) | 2 | 2 | 2 | 2 | 2 | 0 | 2 | 3 | 2 | 17 | moderate risk |
| 81 | Lou et al. 2014 (China, CPIF) | 2 | 2 | 2 | 2 | 2 | 0 | 2 | 3 | 2 | 17 | moderate risk |
| 82 | Lou et al. 2014 (China, PIF) | 2 | 2 | 2 | 2 | 2 | 1 | 2 | 3 | 2 | 18 | moderate risk |
| 83 | Gokmen et al. 2010 (Turkey, PIF) | 2 | 2 | 2 | 2 | 2 | 2 | 2 | 2 | 2 | 18 | moderate risk |
| 84 | Kim et al. 2008 (South Korea, CPIF) | 2 | 2 | 2 | 2 | 2 | 0 | 2 | 3 | 2 | 17 | moderate risk |
| 85 | Jung and Park 2006 (South Korea, PIF) | 2 | 2 | 2 | 2 | 2 | 0 | 2 | 2 | 2 | 16 | moderate risk |
| 86 | Zhao et al. 2010 (China, PIF) | 2 | 2 | 2 | 2 | 2 | 2 | 2 | 3 | 2 | 19 | low risk |
| 87 | Parra et al. 2015 (Chile, PIF) | 2 | 2 | 2 | 2 | 2 | 1 | 2 | 2 | 2 | 17 | moderate risk |
| 88 | El-Sharoud et al. 2008 (Egypt, FUF) | 2 | 2 | 2 | 2 | 2 | 0 | 2 | 2 | 2 | 16 | moderate risk |

Total score <14 = high risk; 14 – 18 = moderate risk; >18 = low risk.

Table S1. Explanatory characteristics of the included data.

| **Characteristic** | **K = 88** |
| --- | --- |
| P | 19.35±33.97 |
| N | 297.07±716.09 |
| Milk_type |  |
| CPIF | 11/88 (13.0%) |
| EPIF | 10/88 (11.0%) |
| FUF | 2/88 (2.3%) |
| IFF | 12/88 (14.0%) |
| Ifoods | 5/88 (5.7%) |
| PMPIF | 48/88 (55%) |
| Method |  |
| C | 19/88 (22.0%) |
| C_API | 25/88 (28.0%) |
| C_API_PCR | 15/88 (17.0%) |
| C_GN_VITEK2 | 1/88 (1.1%) |
| C_GN_VITEK2_PCR | 1/88 (1.1%) |
| C_PCR | 13/88 (15.0%) |
| C_PCR_CPA | 1/88 (1.1%) |
| PCR | 13/88 (15.0%) |
| DNA_extraction |  |
| ANAE | 1/88 (1.1%) |
| Boiling | 11/88 (13.0%) |
| Kit | 24/88 (27.0%) |
| lysis | 2/88 (2.3%) |
| n.a | 46/88 (52%) |
| NS | 4/88 (4.5%) |
| Nation |  |
| Australia | 1/88 (1.1%) |
| Austria | 2/88 (2.3%) |
| Bangladesh | 1/88 (1.1%) |
| Brazil | 5/88 (5.7%) |
| Chile | 2/88 (2.3%) |
| China | 19/88 (22%) |
| Colombia | 1/88 (1.1%) |
| Czech Republic | 4/88 (4.5%) |
| Egypt | 6/88 (6.8%) |
| France | 1/88 (1.1%) |
| Germany | 2/88 (2.3%) |
| India | 1/88 (1.1%) |
| Iran | 3/88 (3.4%) |
| Iraq | 1/88 (1.1%) |
| Ireland | 2/88 (2.3%) |
| Japan | 1/88 (1.1%) |
| Jordan | 3/88 (3.4%) |
| Malaysia | 1/88 (1.1%) |
| Mexico | 2/88 (2.3%) |
| Netherlands | 5/88 (5.7%) |
| Nigeria | 1/88 (1.1%) |
| Slovakia | 4/88 (4.5%) |
| South Africa | 1/88 (1.1%) |
| South Korea | 8/88 (9.1%) |
| Switzerland | 1/88 (1.1%) |
| Turkey | 6/88 (6.8%) |
| UK | 3/88 (3.4%) |
| USA | 1/88 (1.1%) |
| Continent |  |
| Africa | 8/88 (9.1%) |
| Asia | 38/88 (43.0%) |
| Europe | 30/88 (34.0%) |
| North America | 3/88 (3.4%) |
| Oceania | 1/88 (1.1%) |
| South America | 8/88 (9.1%) |
| wb_region |  |
| East Asia and Pacific | 30/88 (34.0%) |
| Europe and Central Asia | 30/88 (34.0%) |
| Latin America and The Caribbean | 10/88 (11.0%) |
| Middle East and North Africa | 13/88 (15.0%) |
| North America | 1/88 (1.1%) |
| South Asia | 2/88 (2.3%) |
| Sub-Saharan Africa | 2/88 (2.3%) |
| wb_income |  |
| High-Income Economies | 37/88 (42%) |
| Lower-Middle Income Economies | 12/88 (14%) |
| Upper-Middle-Income Economies | 39/88 (44%) |
| WHO_region |  |
| African Region (AFR) | 2/88 (2.3%) |
| Eastern Mediterranean Region (EMR) | 13/88 (15%) |
| European Region (EUR) | 30/88 (34%) |
| Region of the Americas (AMR) | 11/88 (13%) |
| South-East Asian Region (SEAR) | 2/88 (2.3%) |
| Western Pacific Region (WPR) | 30/88 (34%) |
| HDI_group |  |
| high HDI | 40/88 (45%) |
| medium HDI | 4/88 (4.5%) |
| very high HDI | 44/88 (50%) |


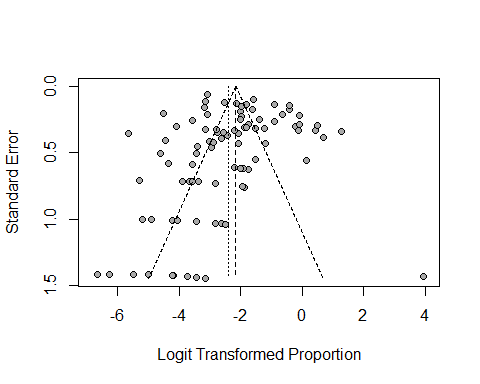


Funnel plot. Eggers' test of the intercept: -0.1, 95% CI; -1.66 - 1.46, t=-0.126, p=0.90.

| **Section and Topic** | **Item #** | **Checklist item** | **Location where item is reported** |
| --- | --- | --- | --- |
| **TITLE** | | |  |
| Title | 1 | Identify the report as a systematic review. | 1 |
| **ABSTRACT** | | |  |
| Abstract | 2 | See the PRISMA 2020 for Abstracts checklist. | 1 |
| **INTRODUCTION** | | |  |
| Rationale | 3 | Describe the rationale for the review in the context of existing knowledge. | 2-4 |
| Objectives | 4 | Provide an explicit statement of the objective(s) or question(s) the review addresses. | 4 |
| **METHODS** | | |  |
| Eligibility criteria | 5 | Specify the inclusion and exclusion criteria for the review and how studies were grouped for the syntheses. | 4-6 |
| Information sources | 6 | Specify all databases, registers, websites, organisations, reference lists and other sources searched or consulted to identify studies. Specify the date when each source was last searched or consulted. | 4-6 |
| Search strategy | 7 | Present the full search strategies for all databases, registers and websites, including any filters and limits used. | Supplementary |
| Selection process | 8 | Specify the methods used to decide whether a study met the inclusion criteria of the review, including how many reviewers screened each record and each report retrieved, whether they worked independently, and if applicable, details of automation tools used in the process. | 4-6 |
| Data collection process | 9 | Specify the methods used to collect data from reports, including how many reviewers collected data from each report, whether they worked independently, any processes for obtaining or confirming data from study investigators, and if applicable, details of automation tools used in the process. | 4-6 |
| Data items | 10a | List and define all outcomes for which data were sought. Specify whether all results that were compatible with each outcome domain in each study were sought (e.g. for all measures, time points, analyses), and if not, the methods used to decide which results to collect. | 4-6 |
|  | 10b | List and define all other variables for which data were sought (e.g. participant and intervention characteristics, funding sources). Describe any assumptions made about any missing or unclear information. | 4-6 |
| Study risk of bias assessment | 11 | Specify the methods used to assess risk of bias in the included studies, including details of the tool(s) used, how many reviewers assessed each study and whether they worked independently, and if applicable, details of automation tools used in the process. | Pg and supplementary |
| Effect measures | 12 | Specify for each outcome the effect measure(s) (e.g. risk ratio, mean difference) used in the synthesis or presentation of results. | 6-7 |
| Synthesis methods | 13a | Describe the processes used to decide which studies were eligible for each synthesis (e.g. tabulating the study intervention characteristics and comparing against the planned groups for each synthesis (item #5)). | 5 and Table 1 in pg 9. |
|  | 13b | Describe any methods required to prepare the data for presentation or synthesis, such as handling of missing summary statistics, or data conversions. | 7 |
|  | 13c | Describe any methods used to tabulate or visually display results of individual studies and syntheses. | 7 |
|  | 13d | Describe any methods used to synthesize results and provide a rationale for the choice(s). If meta-analysis was performed, describe the model(s), method(s) to identify the presence and extent of statistical heterogeneity, and software package(s) used. | 6-7 |
|  | 13e | Describe any methods used to explore possible causes of heterogeneity among study results (e.g. subgroup analysis, meta-regression). | 7 |
|  | 13f | Describe any sensitivity analyses conducted to assess robustness of the synthesized results. | 7 |
| Reporting bias assessment | 14 | Describe any methods used to assess risk of bias due to missing results in a synthesis (arising from reporting biases). | Supplementary |
| Certainty assessment | 15 | Describe any methods used to assess certainty (or confidence) in the body of evidence for an outcome. | 6-7 |
| **RESULTS** | | |  |
| Study selection | 16a | Describe the results of the search and selection process, from the number of records identified in the search to the number of studies included in the review, ideally using a flow diagram. | 8 |
|  | 16b | Cite studies that might appear to meet the inclusion criteria, but which were excluded, and explain why they were excluded. | Supplementary |
| Study characteristics | 17 | Cite each included study and present its characteristics. | Table 1; 9-11 |
| Risk of bias in studies | 18 | Present assessments of risk of bias for each included study. | Supplementary |
| Results of individual studies | 19 | For all outcomes, present, for each study: (a) summary statistics for each group (where appropriate) and (b) an effect estimate and its precision (e.g. confidence/credible interval), ideally using structured tables or plots. | Figure 3, Table 3 |
| Results of syntheses | 20a | For each synthesis, briefly summarise the characteristics and risk of bias among contributing studies. | 11 |
|  | 20b | Present results of all statistical syntheses conducted. If meta-analysis was done, present for each the summary estimate and its precision (e.g. confidence/credible interval) and measures of statistical heterogeneity. If comparing groups, describe the direction of the effect. |  |
|  | 20c | Present results of all investigations of possible causes of heterogeneity among study results. | 11-17; Tables 2 &3 |
|  | 20d | Present results of all sensitivity analyses conducted to assess the robustness of the synthesized results. | 11-17; Tables 2 &3 |
| Reporting biases | 21 | Present assessments of risk of bias due to missing results (arising from reporting biases) for each synthesis assessed. | NA |
| Certainty of evidence | 22 | Present assessments of certainty (or confidence) in the body of evidence for each outcome assessed. | 11 -17; Tables 2 &3 |
| **DISCUSSION** | | |  |
| Discussion | 23a | Provide a general interpretation of the results in the context of other evidence. | 17- 23 |
|  | 23b | Discuss any limitations of the evidence included in the review. | 17- 23 |
|  | 23c | Discuss any limitations of the review processes used. | 17- 23 |
|  | 23d | Discuss implications of the results for practice, policy, and future research. | 23 |
| **OTHER INFORMATION** | | |  |
| Registration and protocol | 24a | Provide registration information for the review, including register name and registration number, or state that the review was not registered. | NA |
|  | 24b | Indicate where the review protocol can be accessed, or state that a protocol was not prepared. | NA |
|  | 24c | Describe and explain any amendments to information provided at registration or in the protocol. | NA |
| Support | 25 | Describe sources of financial or non-financial support for the review, and the role of the funders or sponsors in the review. | NA |
| Competing interests | 26 | Declare any competing interests of review authors. | 24 |
| Availability of data, code and other materials | 27 | Report which of the following are publicly available and where they can be found: template data collection forms; data extracted from included studies; data used for all analyses; analytic code; any other materials used in the review. | 24 |
